# Supplementary material for: Heart-nosed bat alphacoronaviruses use human CEACAM6 to enter cells
Source: Nature. 2026 Apr 22;653(8113):180–9. doi: 10.1038/s41586-026-10394-x (PMC13149331; doi:10.1038/s41586-026-10394-x)
Supplement: Supplementary file 1 — Supplementary Figs. 1–22, Supplementary Tables 1–10 and Supplementary References. [file 41586_2026_10394_MOESM1_ESM.docx]

**Heart-nosed bat alphacoronaviruses use CEACAM6 to enter human cells**

Giulia Gallo^1,2^, Antonello Di Nardo^1^, Doreen Lugano^3^, Adam J. Roberts^4^, Bernadette Ataku Kutima^3^, Moses Okombo^5^, Aghnianditya Kresno Dewantari^1,6^, Florence M. M. Buckley^2^, Gavin J. Wright^4^, James Nyagwange^3^, Bernard Agwanda^7^, Stephen C. Graham^2*^, Dalan Bailey^1*^

^1^ The Pirbright Institute, Ash Road, Pirbright, Surrey GU24 0NF, UK

^2^ Department of Pathology, University of Cambridge, Tennis Court Road, Cambridge CB1 2QP, UK

^3^ KEMRI-Wellcome Trust Research Programme, P.O. Box 230 Kilifi, Kenya

^4^ Hull York Medical School, Department of Biology, York Biomedical Research Institute, University of York, Wentworth Way, York, YO10 5DD, UK

^5^ Loisaba Conservancy, P.O. BOX  1348-10400 Nanyuki, Kenya

^6^ Department of Infectious Disease, Faculty of Medicine, Imperial College London, London SW7 2AZ, UK

^7^ Department of Zoology, National Museum of Kenya, Museum Hill Road, Nairobi

*Co-corresponding authors: contact [scg34@cam.ac.uk](mailto:scg34@cam.ac.uk) and [dalan.bailey@pirbright.ac.uk](mailto:dalan.bailey@pirbright.ac.uk)

**
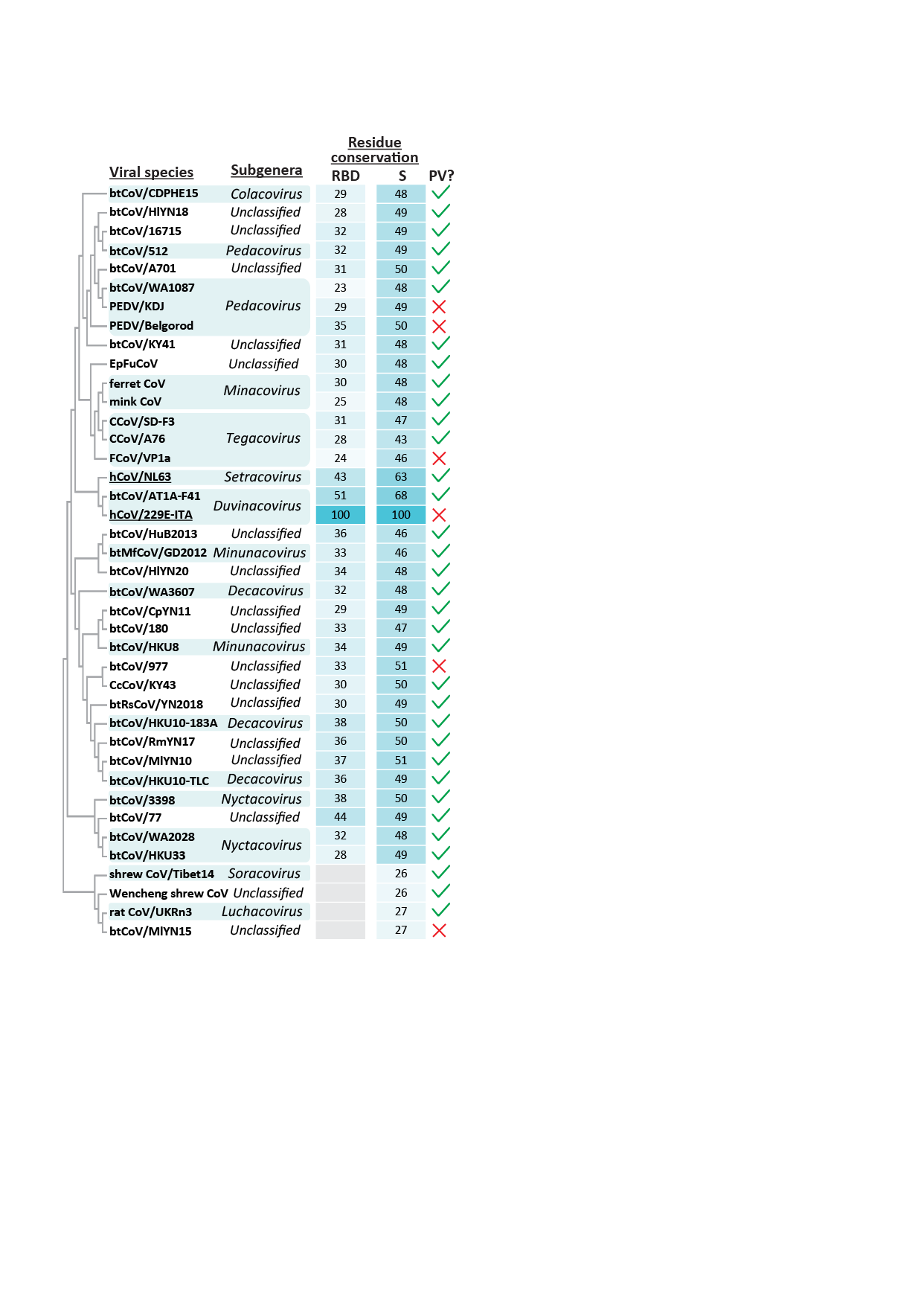
**

**Supplemental Fig.1. Sub-genus classification, amino-acid identity and pseudotyping efficiency of the alphaCoV S library.**

The full-length and predicted RBD of all S proteins in the algorithm-selected alphaCoV library were aligned, with percentage identity to hCoV/299E shown. Where available, the relevant alphaCoV subgenera is also reported. S proteins that could pseudotype are indicated (PV, green tick). For PEDV, feline coronavirus (FCoV), hCoV/229E-ITA and two bat viruses, pseudotypes containing detectable S could not be purified (PV, red cross).


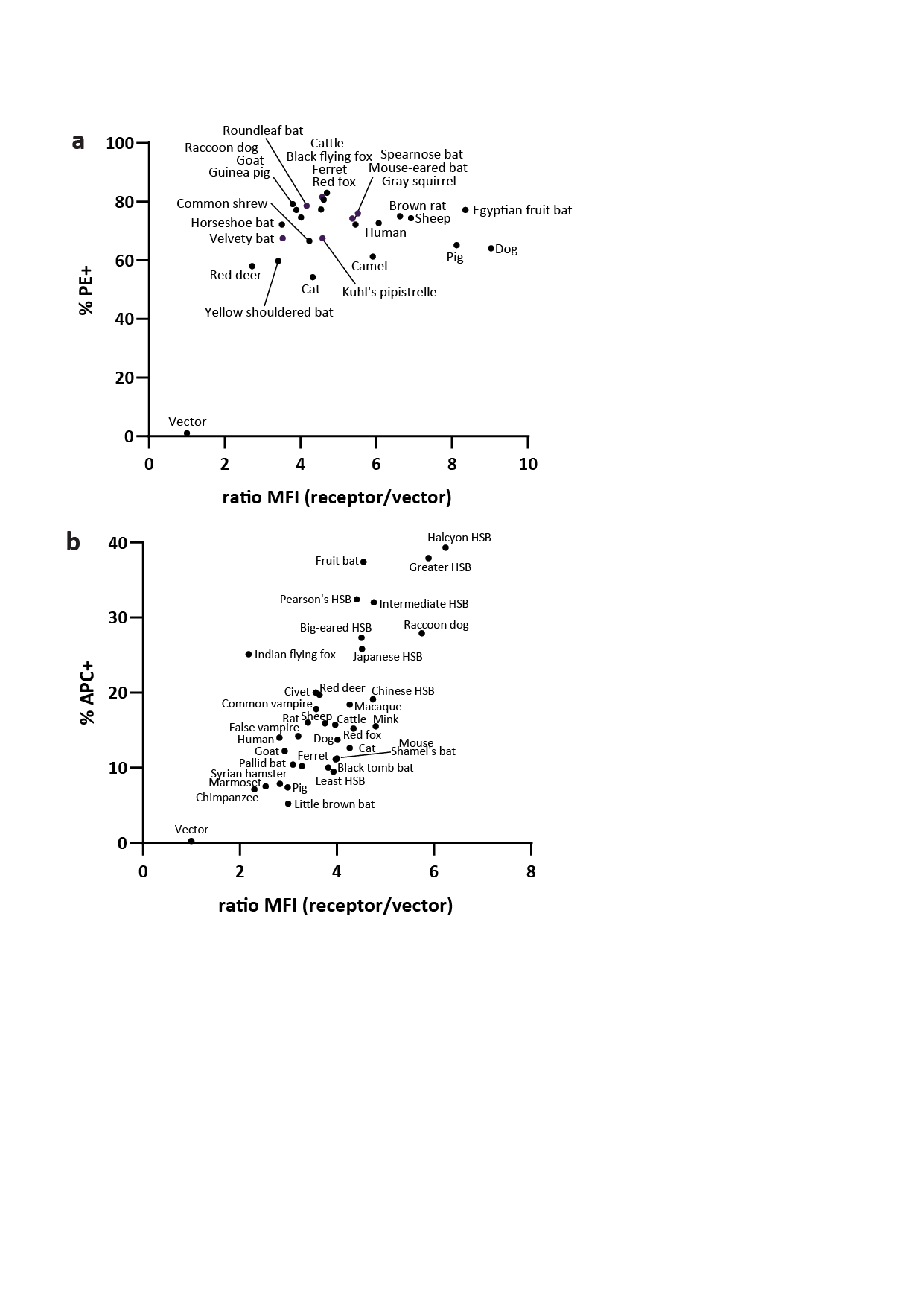


**Supplemental Fig.2. Expression of receptor libraries.**

HEK293T transiently expressing APN (**a**) or ACE2 (**b**) receptors were stained using antibodies against V5 (PE) and FLAG (APC) tags, respectively. Expression was analysed using a MACSQuant cytometer. Data was analysed using FlowJo. Mean fluorescence intensity (MFI) relative to vector alone is plotted against the percentage of positive cells.

**
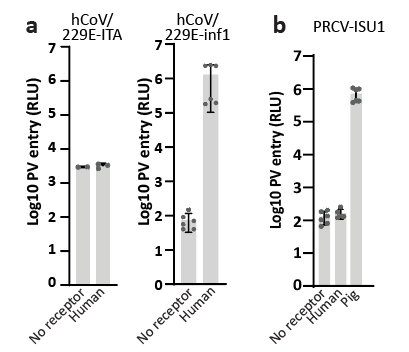
**

**Supplemental Fig.3. hCoV/229E-inf1 and PRCV-ISU1 pseudotyped S can use human and porcine APN, respectively.**

(**a**) Entry assay demonstrating that hCoV/229E (strain: ITA, left panel) pseudotyped S does not enter HEK293T cells over-expressing human APN. 229E-ITA S was subsequently replaced with the S of hCoV/229E (strain: inf-1,) which successfully entered cells overexpressing human APN (right panel). A representative experiment performed in technical triplicates is shown, along with SD. (**b**) Porcine respiratory coronavirus, strain ISU1 (PRCV-ISU1) S pseudotypes can specifically enter HEK293T transiently transfected with a plasmid encoding porcine APN, but not human APN. This is despite the porcine APN receptor not supporting entry for two PEDV strains selected by the greedy algorithm. Raw data from two entry assay performed in technical triplicate is shown, along with SD.


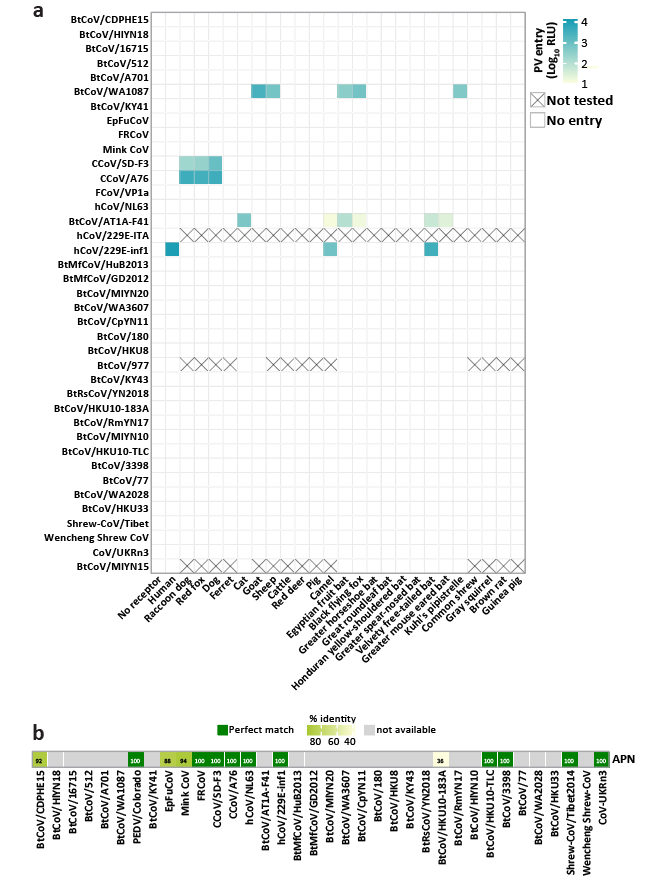


**Supplemental Fig.4. APN receptor screening using the alphaCoV pseudotyped S library.**

(**a**) HEK293T transiently expressing APN from varied mammals were infected with pseudoviruses bearing the indicated alphaCoV S on their surface. Raw pseudovirus (PV) entry data, where background has been subtracted, is shown as log10 relative light units (RLU). Experiments were performed in technical triplicate, and positive results validated with two additional independent experiments. (**b**) For each virus in our library, we report whether the sequence of the APN protein from the respective animal host species is known (numbered boxes) or unknown (grey boxes; NB: either host species was not listed or the APN sequence for a known host species is not available). ‘100’ reflects when a matched APN was used in screening, values <100 reflect the % identity of the closest relative APN used in our screen.

**
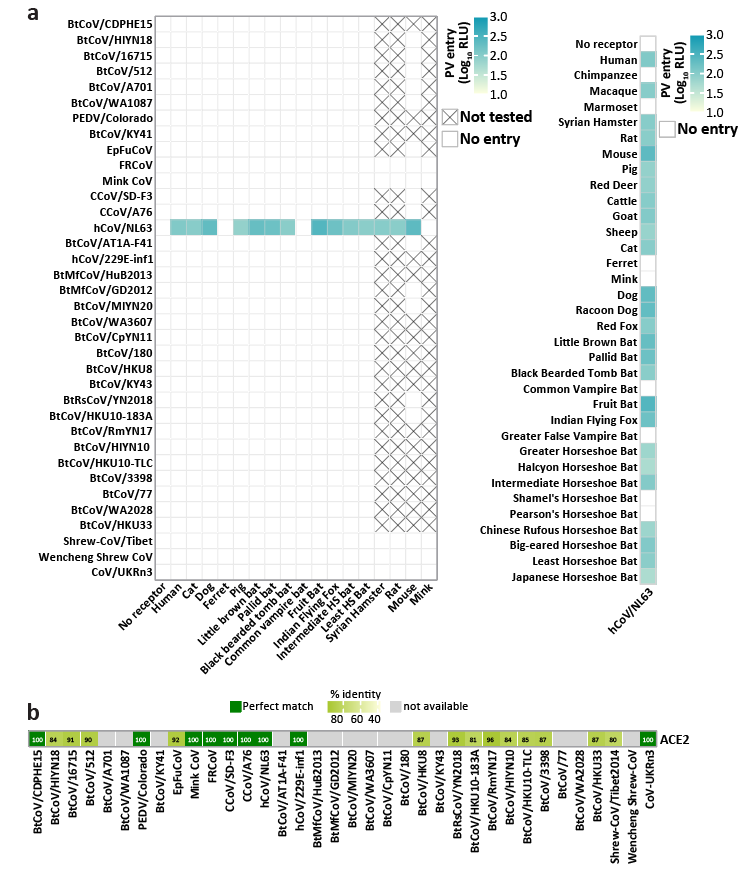
**

**Supplemental Fig.5. ACE2 receptor screening using the alphaCoV pseudotyped S library.**

(a) HEK293T transiently expressing ACE2 from different mammals were infected with pseudoviruses bearing the indicated alphaCoV S on their surface. For hCoV/NL63, a wider panel of ACE2 receptors was screened (as shown). Raw pseudovirus (PV) entry data, where background has been subtracted, is shown as log10 relative light units (RLU). Experiments were performed in technical triplicate, and positive results validated with two additional independent experiments. (**b**) For each virus in our library, we report whether the sequence of the ACE2 protein from the respective animal host species is known (numbered boxes) or unknown (grey boxes; NB: either host species was not listed or the ACE2 sequence for a known host species is not available). ‘100’ reflects when a matched ACE2 was used in screening, values <100 reflect the % identity of the closest relative ACE2 used in our screen.

**
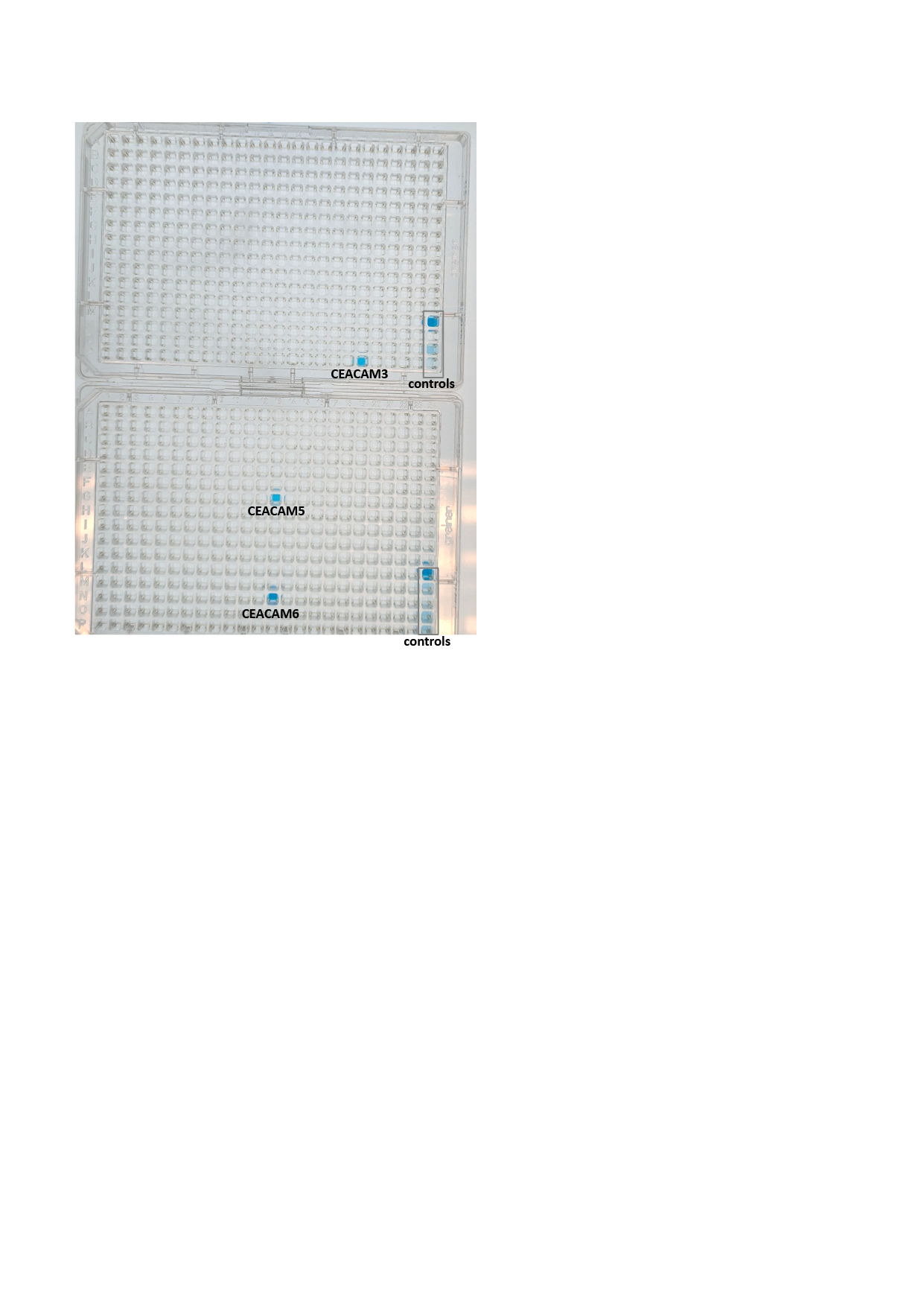
**

**Supplemental Fig.6. Human receptor discovery for CcCoV|KY43 RBD.**

Two 384 well plates containing 759 immobilised human receptor ectodomains were incubated with the CcCoV|KY43 RBD. Protein interactions were visualised using TMB/E solution, stopping the reaction by the addition of NaF to a final concentration 0.15% (w/v) and taking absorbance readings at 652 nm.


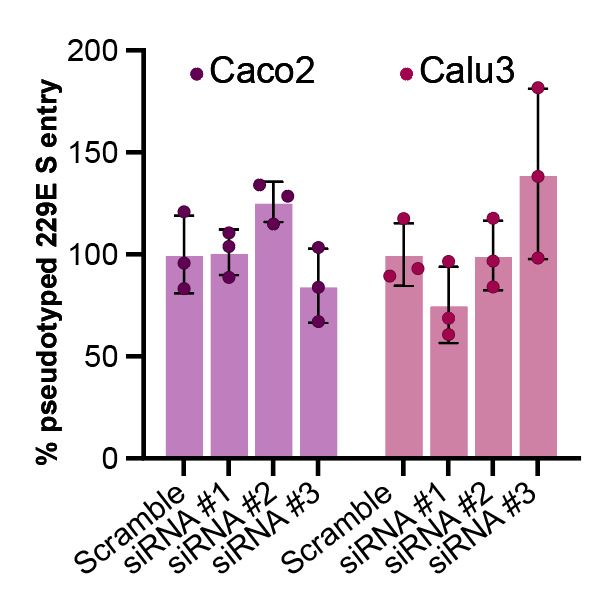


**Supplementary Fig.7. Transient knock-down of human CEACAM6 using siRNA does not affect hCoV/229E entry.** One day after electroporation with different siRNA against human CEACAM6, Caco2 and Calu3 cells (the same used in the experiments described in Figure 2i) were tested for their permissivity to hCoV/229E-inf1, showing no phenotypic effect on entry. Technical triplicates from one experiment are shown.


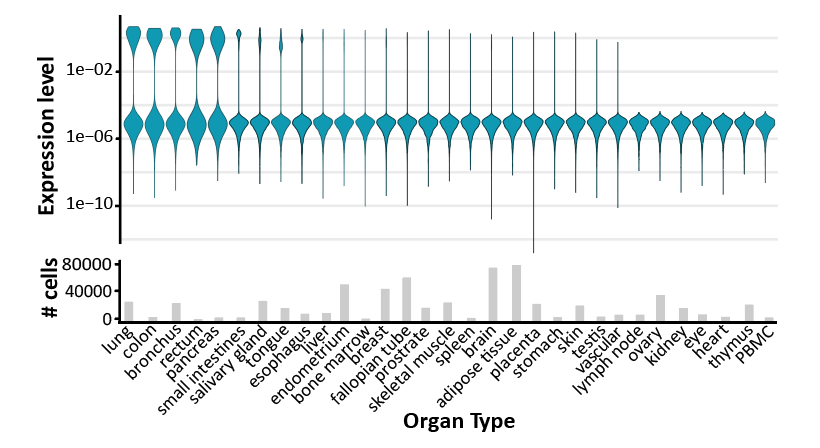


**Supplemental Fig.8. Single cell RNA-sequencing data was retrieved from the Human Protein Atlas.** Relative expression of CEACAM6 per organ is visualized in a violin plot, showing the highest expression in the lung, colon and bronchus. The number of cells for each organ is shown in the separate bar graph below (lung, 27756 cells; colon, 5466 cells; bronchus, 25615 cells).

**
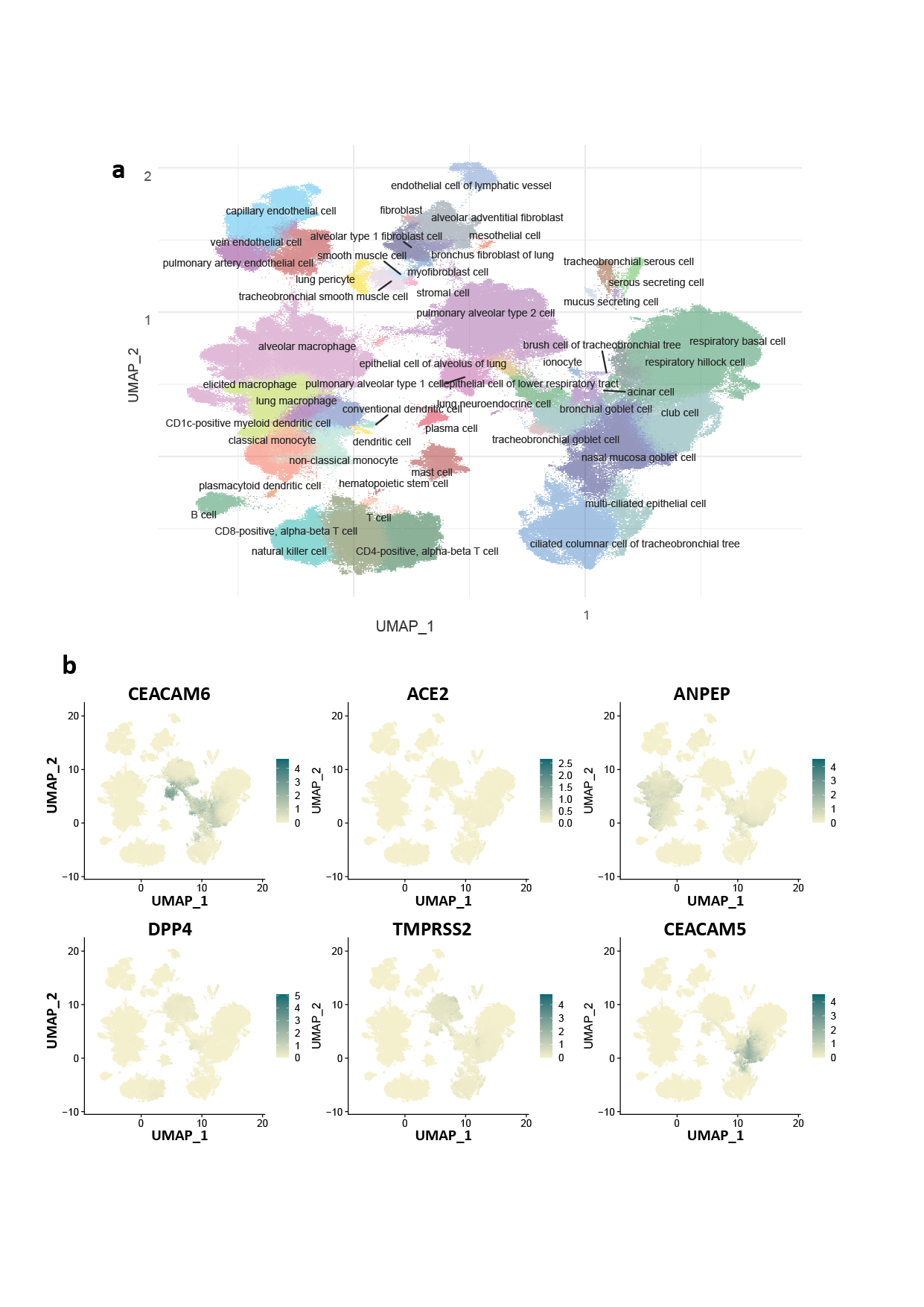
**

**Supplemental Fig.9. UMAPs of annotated lung cell types generated using data from the Human Lung Cell Atlas (HLCA).** (**a**) Cell types found in the lungs were clustered based on gene expression profiles. Each cluster is distinctly coloured and labelled to represent the individual cell types found in the lung. (**b**) Individual UMAPs for CEACAM6, ACE2, ANPEP (APN), DPP4, TMPRSS2 and CEACAM5.

**
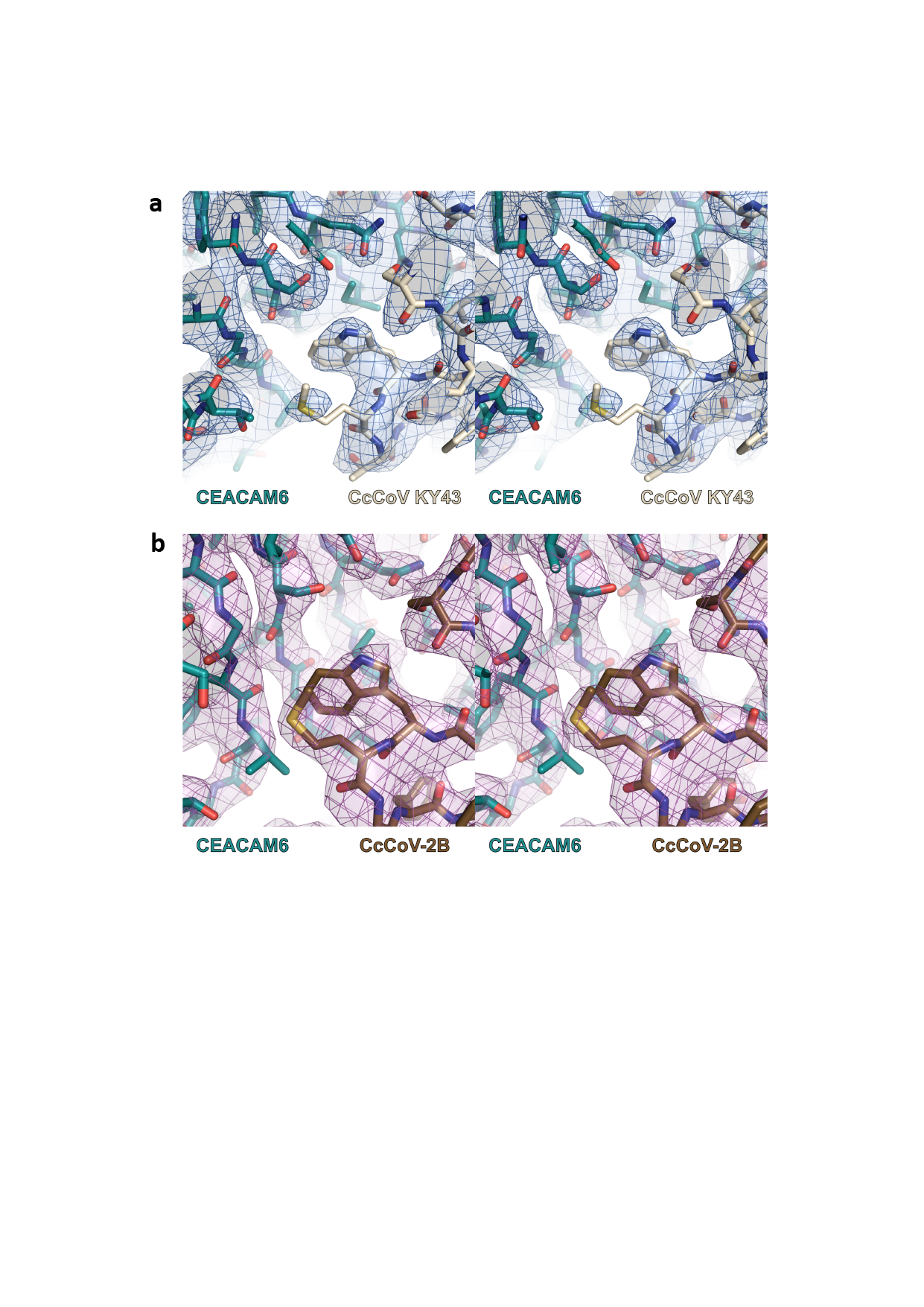
**

**Supplemental Fig.10. Stereo diagrams of final refined CEACAM6 plus RBD crystal structures in 2*F*_O_-*F*_C_ electron density.** CEACAM6 ectodomain is shown in complex with the RBD of (**a**) CcCoV KY43 and (**b**) CcCoV-2B. Electron density was calculated excluding reflections that were not observed experimentally (“no fill” maps) and is shown at 1.2 σ.

**
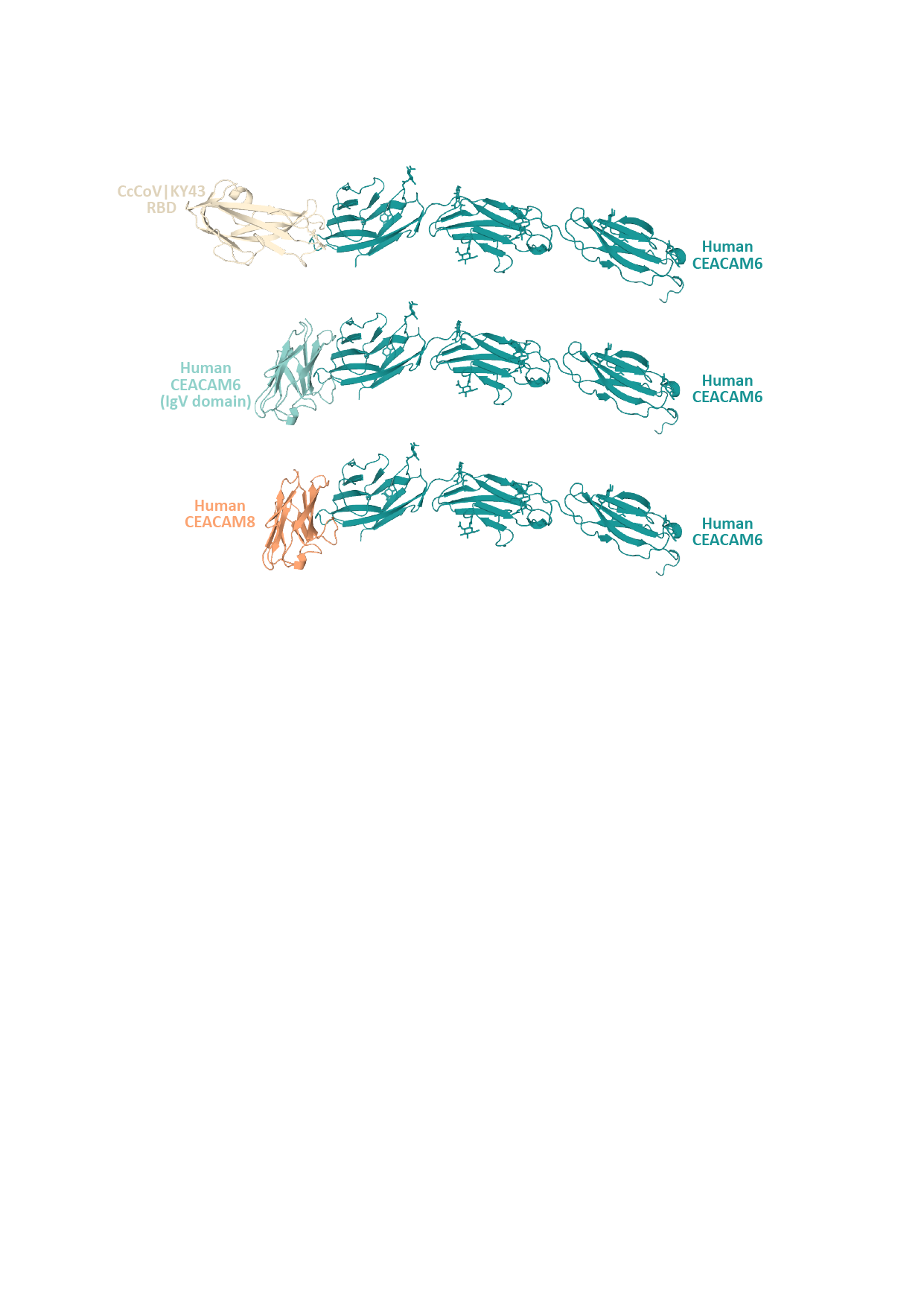
Supplemental Fig.11.Structural comparison of human CEACAM6 homodimer, CEACAM6-CEACAM8 and CEACAM6-KY43 RBD complex.**

CcCoV|KY43 RBD interacts with the N-terminal IgV domain of human CEACAM6, utilising the same interface as the CEACAM6 homodimer (PDB:4Y8A^1^) and the CEACAM6-CEACAM8 heterodimer (PDB:4YIQ^1^).

**
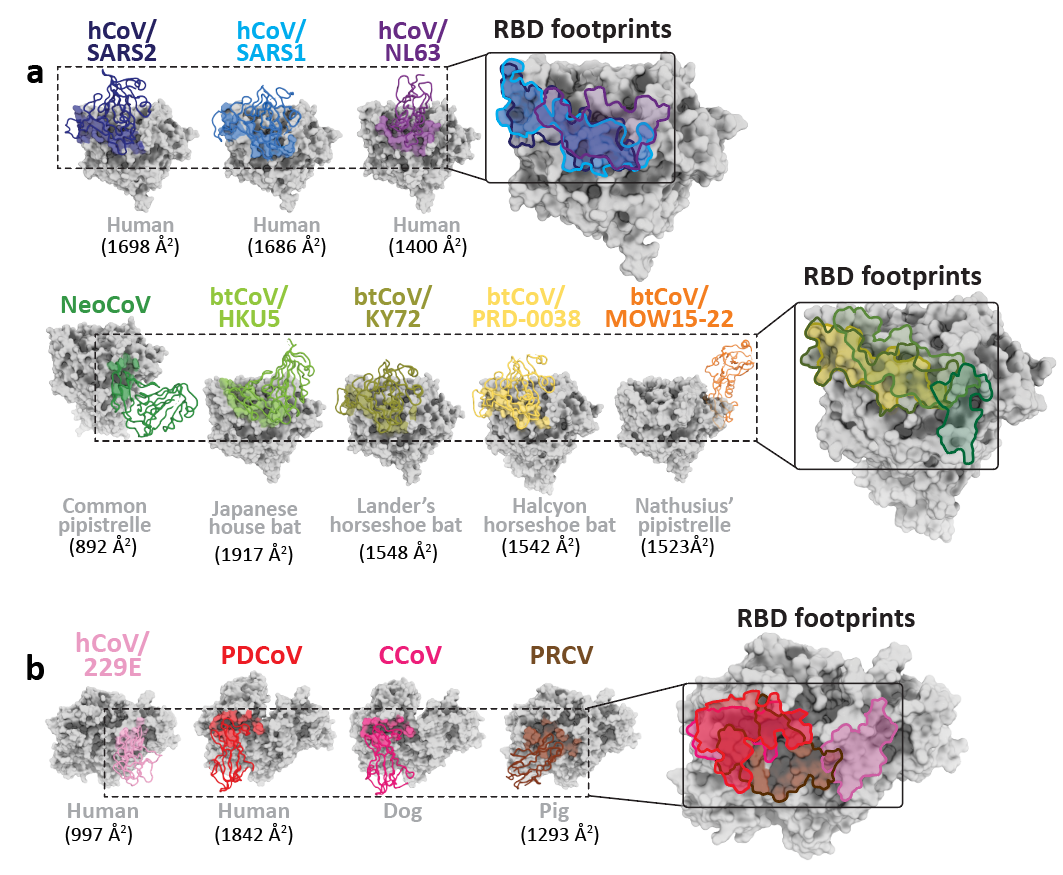
Supplemental Fig.12. Architecture and footprints of experimentally validated coronavirus RBDs-receptors complexes.**

(**a**) The RBDs of coronaviruses interacting with ACE2 from different species are shown. ACE2s have the same orientation, to highlight the different domains used for interaction. Even though viruses bind to different regions, their footprints generally overlap, with the exception of btCoV/MOW15-22 (PDB: 9C6O^2^), whose interface, on the opposite side of ACE2, is completely distinct from those of other viruses (hCoV/SARS2, PDB: 6M0J^3^; hCoV/SARS1, PDB: 2AJF^4^; NeoCoV, PDB: 7WPO^5^; btCoV/HKU5, PDB: 9D32^6^; btCoV/KY72, PDB: 8K4U^7^; btCoV/PRD-0038, PDB: 8U0T^8^). Of note, hCoV/NL63, the only alphaCoV using ACE2 (PDB: 3KBH^9^), shares a similar interface to the other betacoronaviruses, despite the architecture of its RBD being different. (**b**) Binding footprint of alphaCoV RBDs on APN. There is relatively little overlap in the regions of APN used by alphacoronaviruses hCoV/229E (PDB: 6ATK^10^), canine CoV (CCoV strain HuPn2018, PDB: 7U0L^11^) and porcine coronavirus PRCV (PDB: 4F5C^12^). Despite belonging to a different genus, porcine deltacoronavirus (PDCoV) can also employ human and porcine APN to enter cells (PDB: 7VPQ^13^), with a binding footprint similar to canine CoV. Total buried surface area is indicated below each complex.


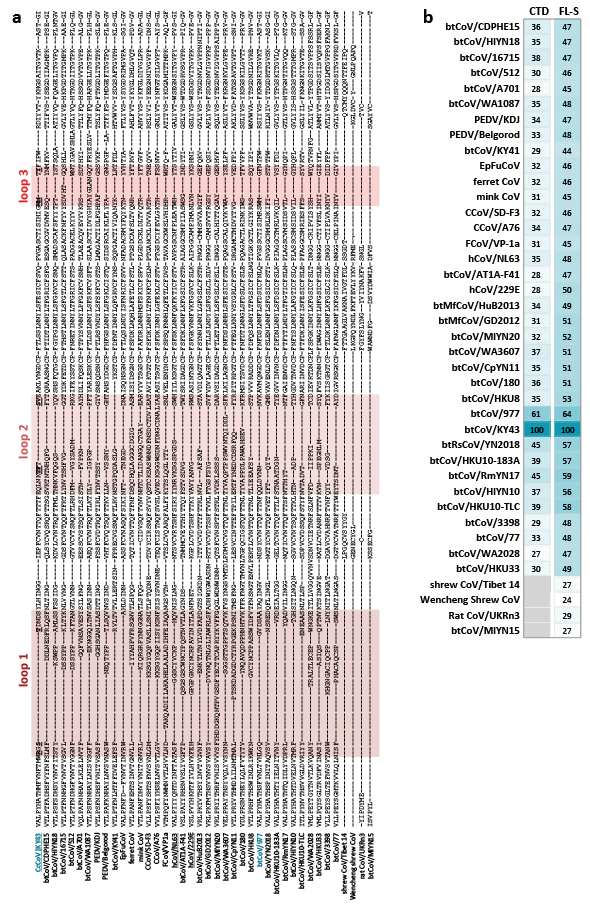


**Supplemental Fig.13. Comparison of CcCoV|KY43 S amino acid sequence with the alphaCoV library.** (**a**) the RBD of CcCoV|KY43 aligned with the predicted RBD from the other alphaCoV S in the algorithm-selected library. Loops involved in the interaction with human CEACAM6 are highlighted, showing low conservation. (**b**) Percentage amino acid identities between the S and RBDs of the library alphaCoVs and CcCoV|KY43 are shown. btCoV/977, which also uses CEACAM6 as receptor, shares only 61% amino acid identity with CcCoV|KY43.

**
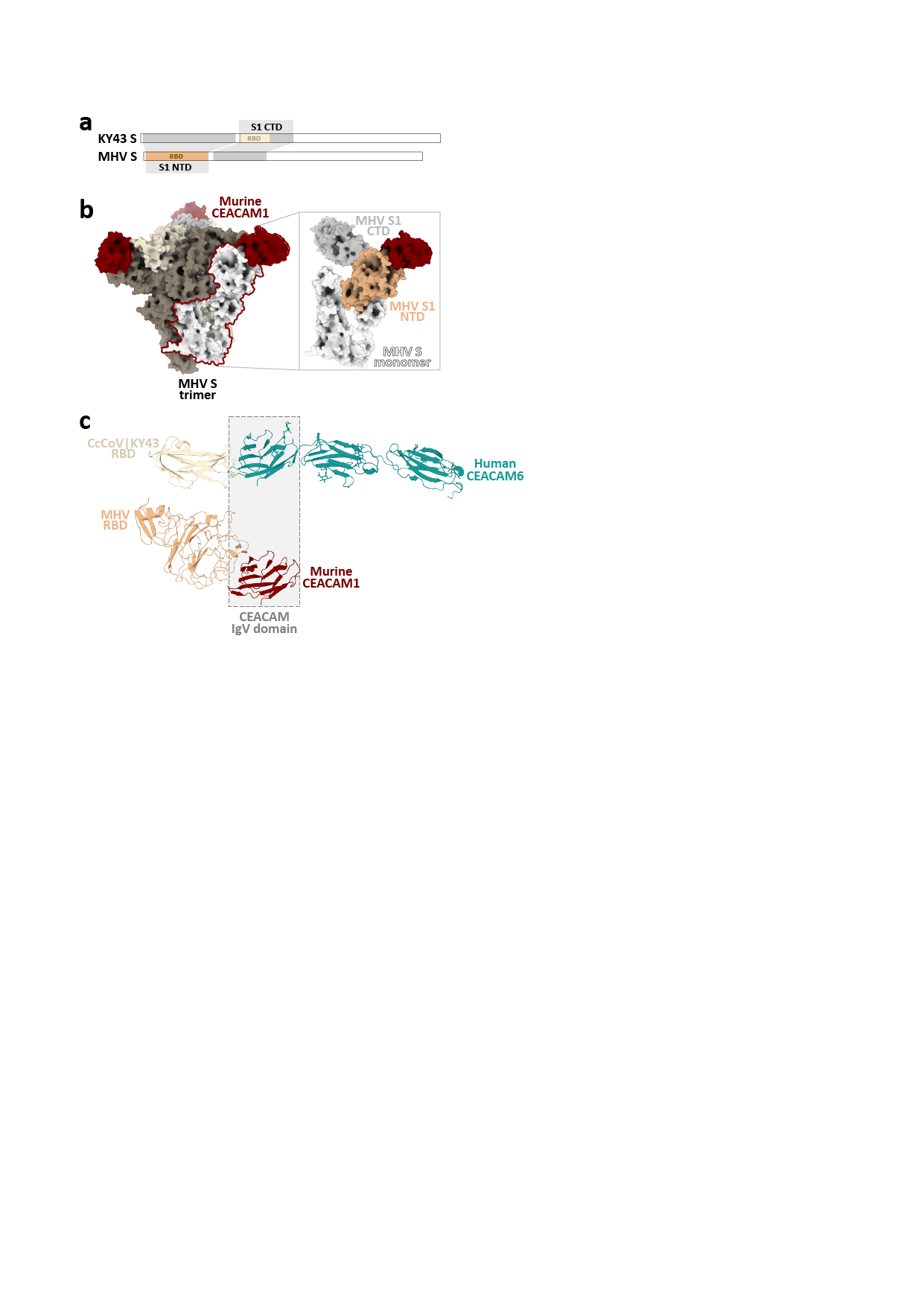
Supplemental Fig.14. The MHV Spike N-terminal domain interacts with its murine receptor CEACAM1.**

(**a**) Graphical alignment of CcCoV|KY43 and MHV S. Their respective NTDs and CTDs are shown, highlighting the differing location of the protein-binding RBD. (**b**) Cryo-EM structure of a MHV S trimer in complex with murine CEACAM1 (PDB:6VSJ^14^). In the inset, an S monomer is depicted with coloured NTD (pale orange) and CTD (dark grey), highlighting the interaction of the receptor with the S1 NTD. (**c**) Comparison of CEACAM1 and CEACAM6 N-terminal IgV domains. Even though CcCoV|KY43 and MHV belong to different coronavirus genera and the organization of their RBD is not conserved, both S bind to the same CEACAM domain, albeit at different interfaces.


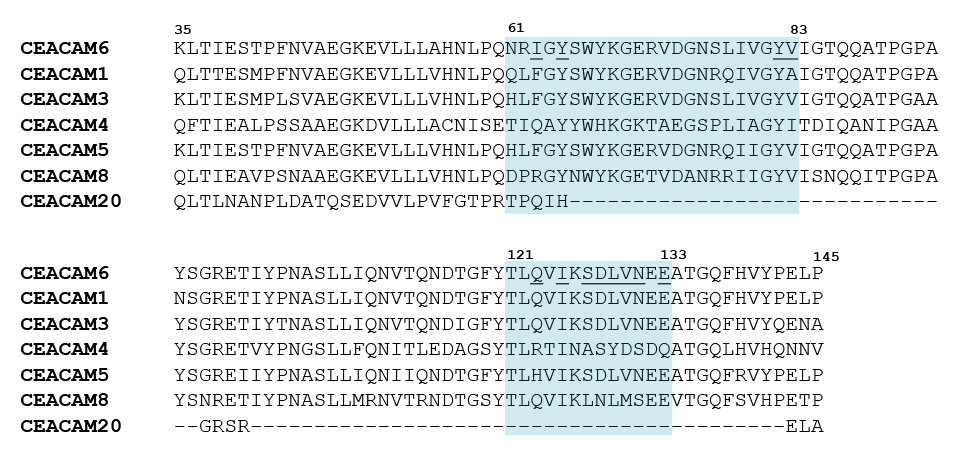


**Supplemental Fig.15. Sequence comparison of the amino-terminal V-type Ig-like domain of human CEACAMs.** The alignment shows high conservation of the CcCoV|KY43 binding site; however, human CEACAM6 is the only protein with an isoleucine at position 63, which is a key determinant of receptor usage. Residues involved in the interaction with CcCoV|KY43 RBD are underlined. The percentage amino acid identity of each domain with CEACAM6 is as follows: CEACAM1, 90.1%; CEACAM3, 88.3%; CEACAM4, 48.6%; CEACAM5, 89.2%; CEACAM8, 72.1%; CEACAM20, 26.3%.

**
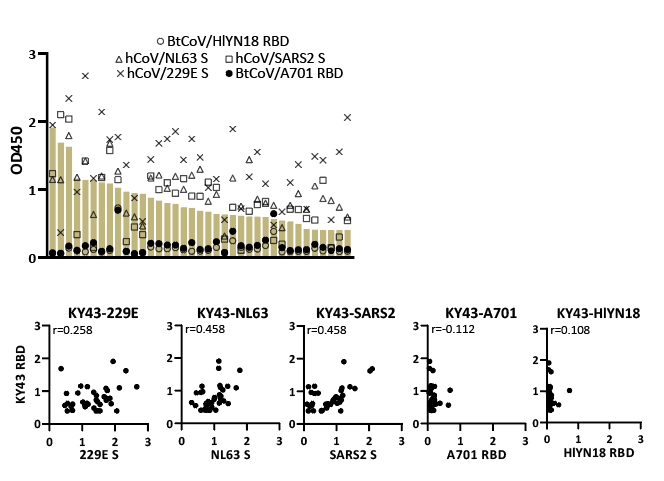
**

**Supplemental Fig.16. Correlation of high CcCoV|KY43 ELISA reactivity with responses to other coronavirus antigens. Top:** ELISA optical density (OD) values for all samples within the upper 10% of the KY43 distribution. **Below:** Pearson’s correlation analysis was performed on this enriched subset of ‘high’ KY43 samples to assess co-variation. Each point represents an individual serum sample tested against matched antigens. Correlation coefficients (r) are shown for each comparison. Of note, the top 10% threshold was used as a descriptive enrichment criterion and not to infer seropositivity or compare absolute signal magnitudes across assays.


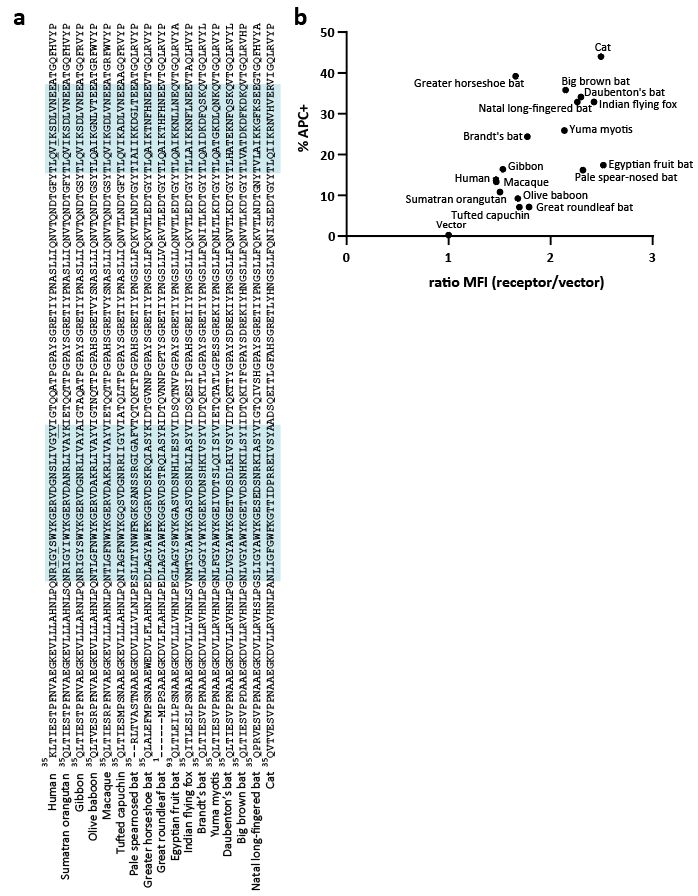


**Supplemental Fig.17. Alignment and expression of the CEACAM6 receptor library.**

(**a**) Sequences of the IgV domain from the CEACAM6s included in our library are shown. Domains responsible for the binding to CcCoV|KY43 are highlighted in teal, while residues in direct contact with the CcCoV RBDs are underlined. (**b**) HEK293T transiently expressing the indicated CEACAM6 protein were stained using an antibody against the appended His tag, coupled to an APC fluorochrome. Expression was analysed using MACSQuant cytometer. Data was analysed using FlowJo. Mean fluorescence intensity (MFI) relative to vector alone is plotted against the percentage of APC positive cells (%APC+).


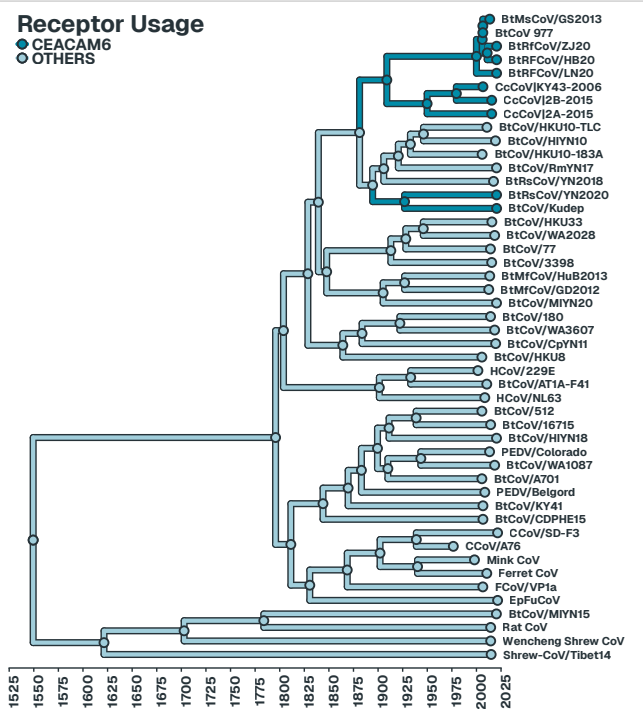


**Supplemental Fig.18. Evolutionary acquisition of CEACAM6 usage among alphaCoVs**. Time-scaled Highest Independent Posterior Subtree (HIPSTR) phylogeny inferred in BEAST 1.10.5 [REF] under a binary CTMC model of receptor usage with Markov jump counting. Branches and nodes are coloured by the posterior most probable state (CEACAM6 usage versus OTHER receptor usage), summarizing inferred trait history across the posterior distribution.

**
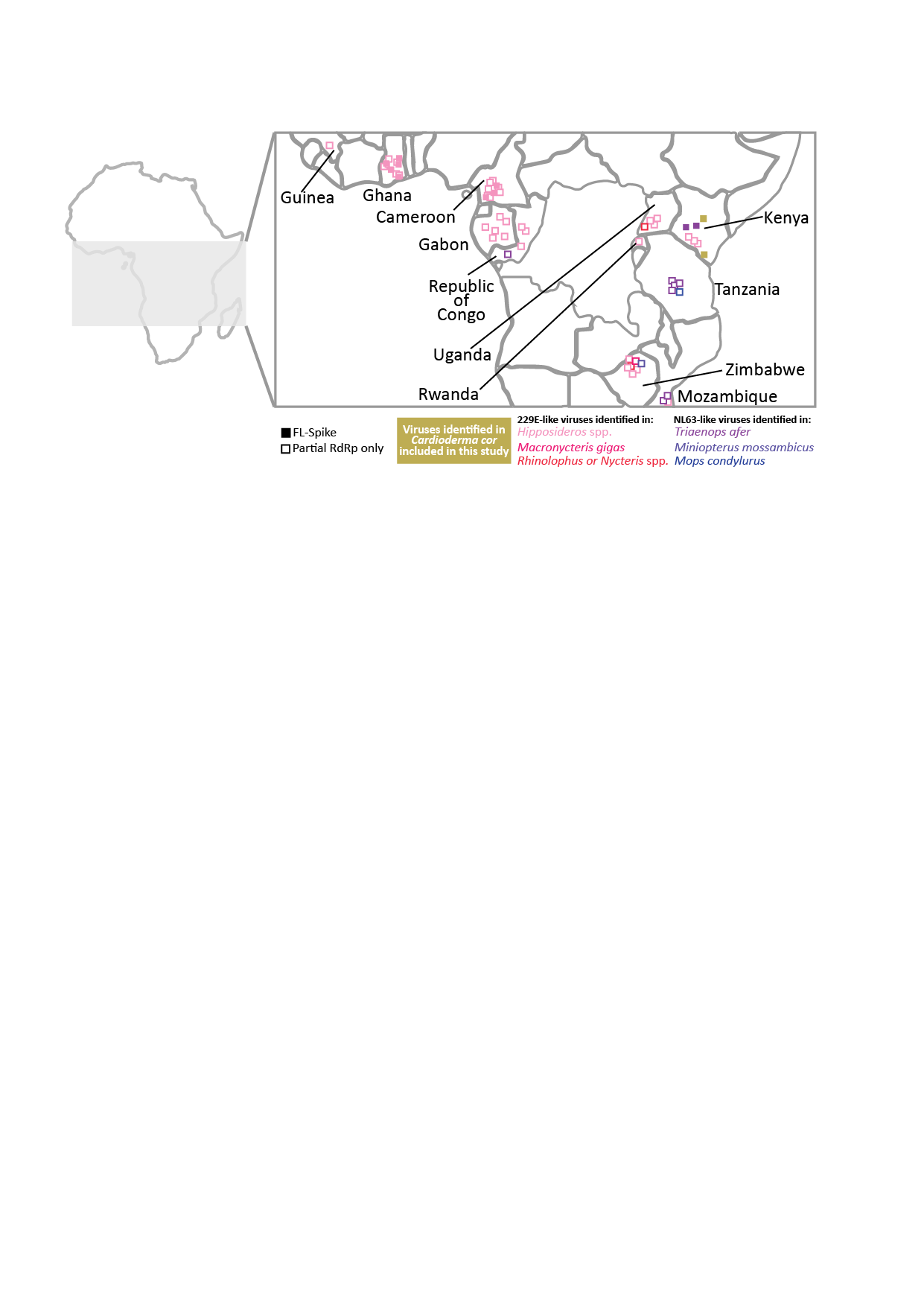
 Supplemental Fig.19. Bat 229E-like and NL63-like viruses have been identified in sub-Saharan Africa, where CcCoVs also circulate.**

AlphaCoVs closely related to the endemic human coronaviruses 229E (shaded, pink/red) and NL63 (shaded, purple/blue) were found in various bat populations by sequencing (Spike or RdRp; full or empty squares, respectively). The CcCoV included in this study are found within the same geographical region (mustard squares, Kenya).

**
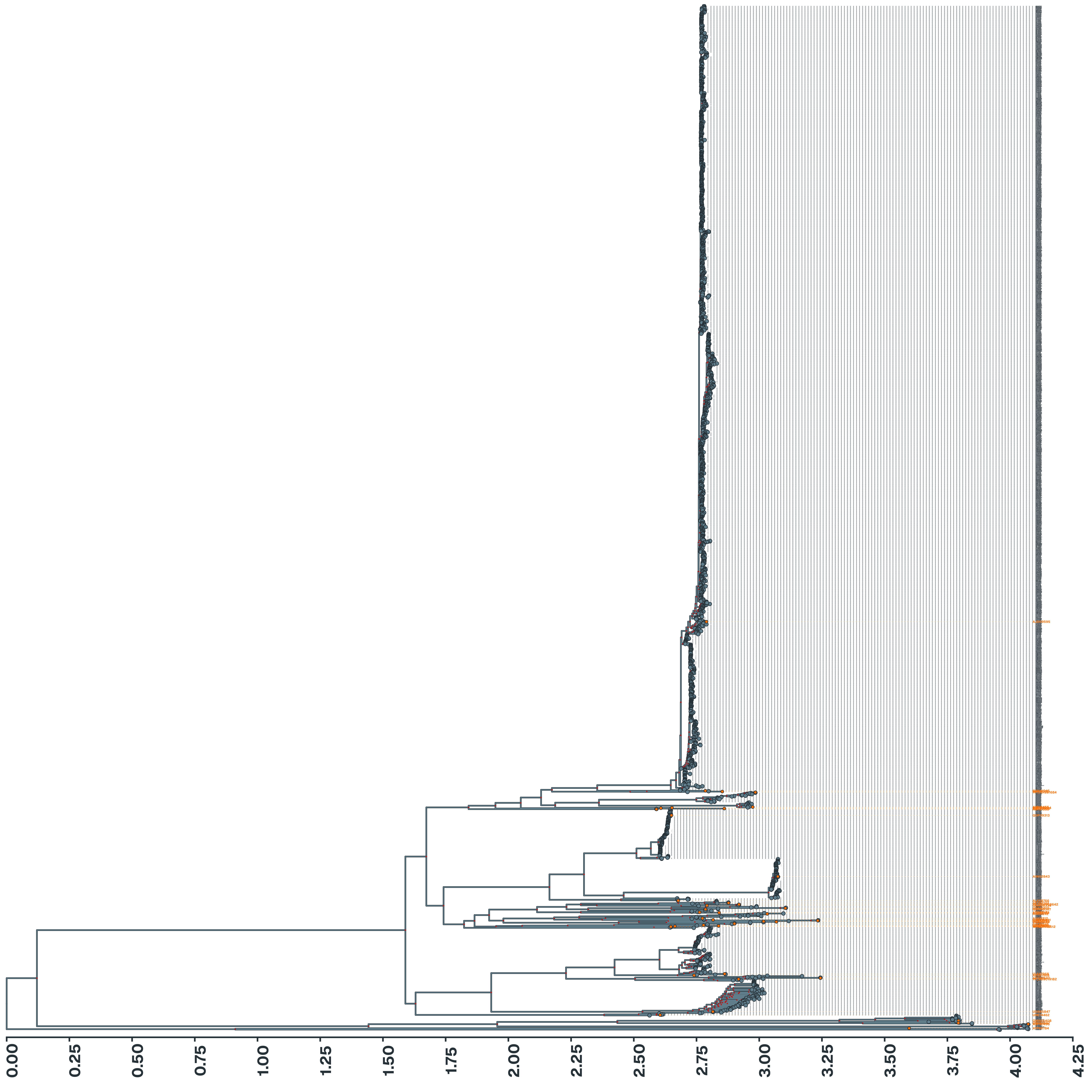
**

**Supplemental Fig.20. Global diversity of alphacoronavirus spike genes and coverage of the experimental panel.**

Maximum-likelihood phylogeny inferred with IQ-TREE 2.3.4 from 2,714 full-length spike coding sequences, retrieved from the Virus Pathogen Database and Analysis Resource (ViPR) platform, under a codon-partitioned substitution model. The 40 greedily selected spike sequences used for functional assays are indicated by orange tip labels. Branch lengths are in units of expected substitutions per site on the x-axis, as estimated by IQ-TREE.

**
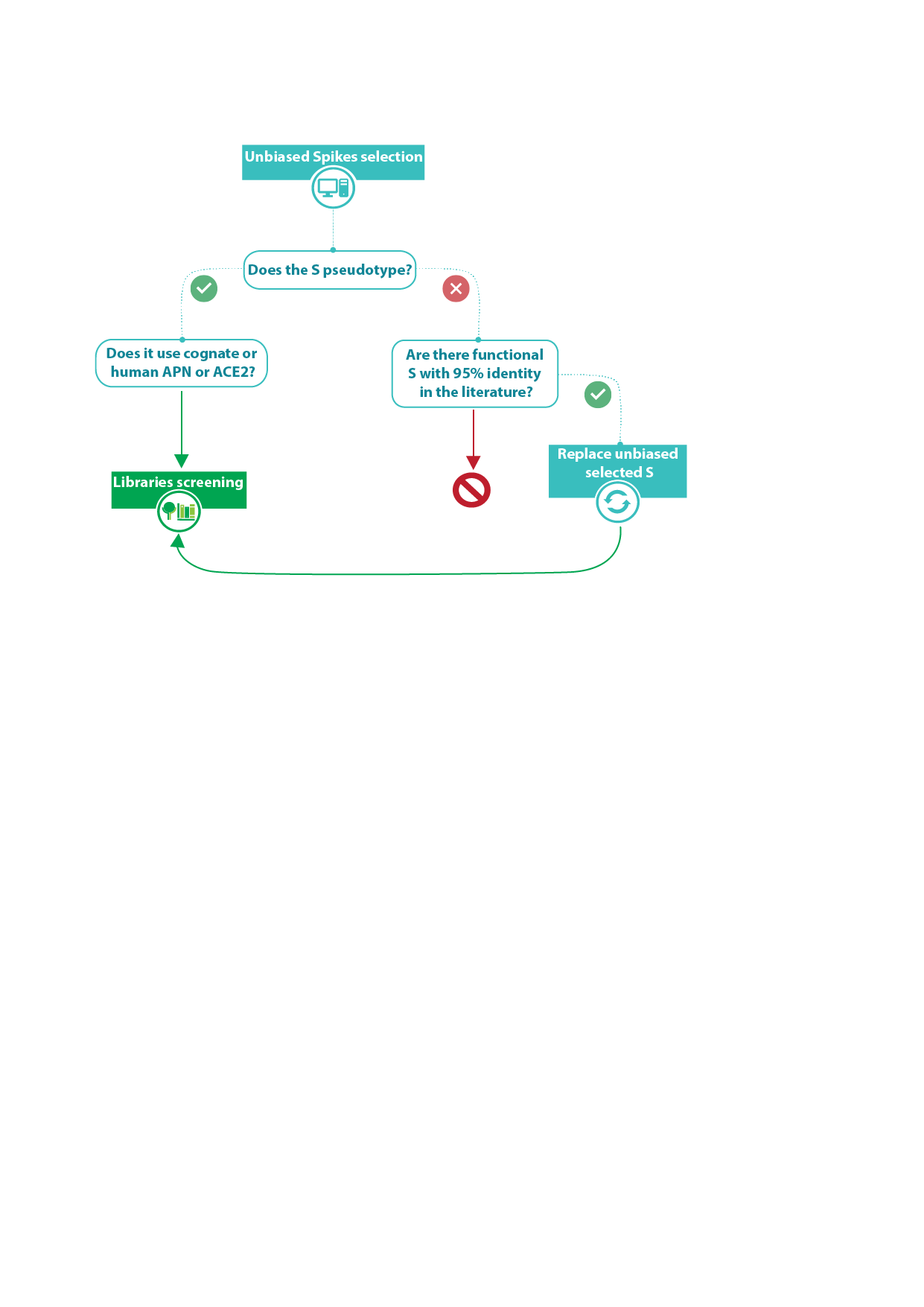
Supplemental Fig.21. Selection pipeline for alternative S proteins.**

Sequences selected by the greedy algorithm were assessed for their pseudotyping efficiency. All functional S were used for screening of APN and ACE2 receptor usage. When no S could be detected by either immunoblot or in entry assays, a related sequence was used as a replacement, but only if it shared >95% amino acid identity and was reported to be a functional APN or ACE2 receptor user in the literature (e.g. PEDV/Colorado).


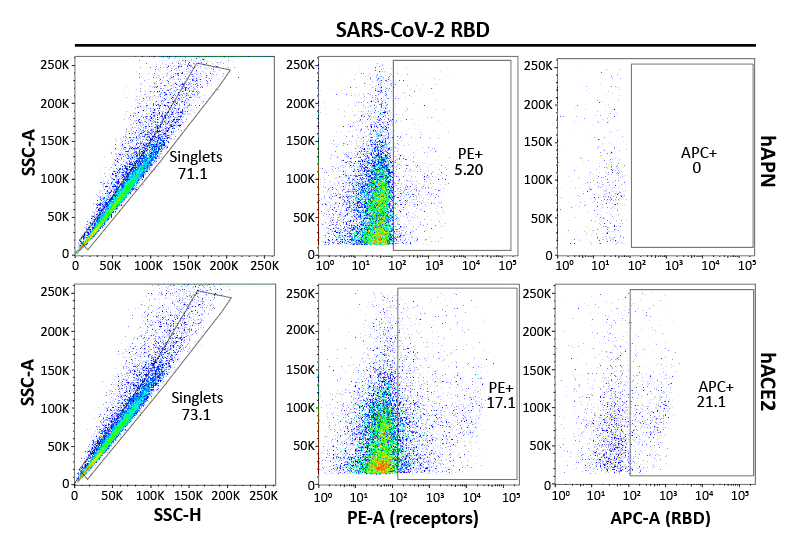


**Supplementary Fig.22. Gating strategy used for flow cytometry.**

HEK293T transiently expressing the receptors (PE positive recognizing either V5 for the APN library, HA for ACE2 library and hDPP4, Flag tag for hTMPRSS2 and hDPEP1) were incubated with the His-tagged RBDs (APC positives). SARS-CoV-2, known to use human ACE2 but not human APN, was used for gating.

**References for Supplementary Figures:**

1. Bonsor, D.A., Günther, S., Beadenkopf, R., Beckett, D. & Sundberg, E.J. Diverse oligomeric states of CEACAM IgV domains. *Proc Natl Acad Sci U S A* **112**, 13561-13566 (2015).

2. Ma, C.B. *et al.* Multiple independent acquisitions of ACE2 usage in MERS-related coronaviruses. *Cell* **188**, 1693-1710.e1618 (2025).

3. Lan, J. *et al.* Structure of the SARS-CoV-2 spike receptor-binding domain bound to the ACE2 receptor. *Nature* **581**, 215-220 (2020).

4. Li, F., Li, W., Farzan, M. & Harrison, S.C. Structure of SARS coronavirus spike receptor-binding domain complexed with receptor. *Science* **309**, 1864-1868 (2005).

5. Xiong, Q. *et al.* Close relatives of MERS-CoV in bats use ACE2 as their functional receptors. *Nature* **612**, 748-757 (2022).

6. Park, Y.J. *et al.* Molecular basis of convergent evolution of ACE2 receptor utilization among HKU5 coronaviruses. *Cell* **188**, 1711-1728.e1721 (2025).

7. Su, C. *et al.* Structural characteristics of BtKY72 RBD bound to bat ACE2 reveal multiple key residues affecting ACE2 usage of sarbecoviruses. *mBio* **15**, e0140424 (2024).

8. Lee, J. *et al.* Broad receptor tropism and immunogenicity of a clade 3 sarbecovirus. *Cell Host Microbe* **31**, 1961-1973.e1911 (2023).

9. Wu, K., Li, W., Peng, G. & Li, F. Crystal structure of NL63 respiratory coronavirus receptor-binding domain complexed with its human receptor. *Proc Natl Acad Sci U S A* **106**, 19970-19974 (2009).

10. Wong, A.H.M. *et al.* Receptor-binding loops in alphacoronavirus adaptation and evolution. *Nat Commun* **8**, 1735 (2017).

11. Tortorici, M.A. *et al.* Structure, receptor recognition, and antigenicity of the human coronavirus CCoV-HuPn-2018 spike glycoprotein. *Cell* **185**, 2279-2291.e2217 (2022).

12. Reguera, J. *et al.* Structural bases of coronavirus attachment to host aminopeptidase N and its inhibition by neutralizing antibodies. *PLoS Pathog* **8**, e1002859 (2012).

13. Ji, W. *et al.* Structures of a deltacoronavirus spike protein bound to porcine and human receptors. *Nat Commun* **13**, 1467 (2022).

14. Shang, J. *et al.* Structure of mouse coronavirus spike protein complexed with receptor reveals mechanism for viral entry. *PLoS Pathog* **16**, e1008392 (2020).

**Table S1. Accession numbers of the Spikes of the alphaCoV library.**

| ID | GenBank accession number |
| --- | --- |
| BtCoV/CDPHE15 | AGT21333 |
| BtCoV/HlYN18 | QWN56394 |
| BtCoV/16715 | AYR18455 |
| BtCoV/512 | YP-001351684 |
| BtCoV/A701 | ABG11965 |
| BtCoV/WA1087 | QGX41945 |
| PEDV/KDJ | AJD09595 |
| PEDV/Colorado | AGO58924.1 |
| PEDV/Belgorod | ASV51733 |
| BtCoV/KY41 | ADX59458 |
| EpFuCoV | UNE74462 |
| FRCoV | AKG92640 |
| Mink CoV | ADI80513 |
| CCoV/SD-F3 | ULF47968 |
| CCoV/A76 | AEQ61968 |
| FCoV/VP1a | UOS85847 |
| hCoV/NL63 | AIW52843 |
| BtCoV/AT1A-F41 | ALK28766 |
| hCoV/229E-ITA | QOP39313 |
| hCoV/229E-inf-1 | NP_073551.1 |
| BtMfCoV/HuB2013 | AIA62242 |
| BtMfCoV/GD2012 | AIA62212 |
| BtCoV/MlYN20 | QWN56401 |
| BtCoV/WA3607 | QGX41951 |
| BtCoV/CpYN11 | QWN56273 |
| BtCoV/180 | UIG55624 |
| BtCoV/HKU8 | ACA52171 |
| BtCoV/977 | ABO88151 |
| BtCoV/KY43 | ADX59451 |
| BtRsCoV/YN2018 | QDF43810 |
| BtCoV/HKU10-183A | YP-006908642 |
| BtCoV/RmYN17 | QWN56367 |
| BtCoV/MlYN10 | QWN56334 |
| BtCoV/HKU10-TLC | AFU92122 |
| BtCoV/3398 | YP-009755890 |
| BtCoV/77 | ULD45286 |
| BtCoV/WA2028 | QGX41957 |
| BtCoV/HKU33 | QCX35160 |
| Shrew-CoV/Tibet14 | ATP66784 |
| Wencheng Shrew-CoV | ASF90496 |
| Rat/CoV-UKRn3 | QBG64657 |
| BtCoV/MlYN15 | QWN56408 |
| PRCV-ISU1 | ABG89317 |

**Table S2. Accession numbers of the APN library.**

| Common name | Scientific name | Genbank accession number |
| --- | --- | --- |
| Human | *Homo sapiens* | NP_001141.2 |
| Raccoon dog | *Nyctereutes procyonoides* | XP_055182557.1 |
| Red fox | *Vulpes vulpes* | XP_025856768.1 |
| Dog | *Canis lupus familiaris* | NP_001139506.1 |
| Ferret | *Mustela putorius furo* | XP_012917463.1 |
| Cat | *Felis catus* | NP_001009252.2 |
| Goat | *Capra hircus* | XP_005695088.3 |
| Sheep | *Ovis aries* | XP_014957374.3 |
| Cattle | *Bos taurus* | NP_001068612.1 |
| European red deer | *Cervus elaphus* | OWK08899.1 |
| Pig | *Sus scrofa* | XP_005653580.1 |
| Bactrian camel | *Camelus ferus* | XP_006192640.2 |
| Egyptian fruit bat | *Rousettus aegypticus* | XP_016007055.1 |
| Black flying fox | *Pteropus alecto* | XP_006905412.1 |
| Greater horseshoe bat | *Rhinolophus ferrumequinum* | XP_032956109.1 |
| Great roundleaf bat | *Hipposideros armiger* | XP_019495552.1 |
| Honduran yellow-shouldered bat | *Sturnira hondurensis* | XP_036909514.1 |
| Greater spear-nosed bat | *Phyllostomus hastatus* | XP_045680750.1 |
| Velvety free-tailed bat | *Molossus molossus* | XP_03613394.1 |
| Greater mouse-eared bat | *Myotis myotis* | XP_036211683.1 |
| Kuhl’s pipistrelle | *Pipistrellus kuhlii* | XP_36271433.1 |
| Common shrew | *Sorex araneus* | XP_004617569.1 |
| Gray squirrel | *Sciurus carolinensis* | MBZ3880029.1 |
| Brown rat | *Rattus norvegicus* | NP_112274.1 |
| Guinea pig | *Cavia porcellus* | XP_003475291.1 |

T**able S3. Accession numbers of the ACE2 library.**

| Common name | Scientific name | Genbank accession number |
| --- | --- | --- |
| Human | *Homo sapiens* | BAB40370.1 |
| Chimpanzee | *Pan troglodytes* | XP_016798468.1 |
| Rhesus macaque | *Macaca mulatta* | ACI04575.1 |
| Marmoset | *Callithrix jacchus* | XP_008987241.1 |
| Golden hamster | *Mesocricetus auratus* | XP_005074266.1–– |
| Brown rat | *Rattus norvegicus* | NP_001012006.1 |
| House mouse | *Mus musculus* | NP_001123985.1 |
| Pig | *Sus scrofa* | NP_001116542.1 |
| Red deer | *Cervus elaphus* | XP_043752042.1 |
| Cattle | *Bos taurus* | NP_001019673.2 |
| Goat | *Capra hircus* | AHI85757.1 |
| Sheep | *Ovis aries* | XP_011961657.1 |
| Cat | *Felis catus* | AAX59005.1 |
| Ferret | *Mustela putorius furo* | BAE53380.1 |
| Mink | *Neogale vison* | XP_044091953.1 |
| Dog | *Canis lupus familiaris* | ACT66277.1 |
| Raccoon dog | *Nyctereutes procyonoides* | ABW16956.1 |
| Red fox | *Vulpes vulpes* | XP_025842513.1 |
| Little Brown Bat | *Myotis lucifugus* | XP_023609438.1 |
| Pallid bat | *Antrozous pallidus* | QJF77789.1 |
| Black-bearded tomb bat | *Taphozous melanopogon* | UJP38391.1 |
| Common vampire bat | *Desmodus rotundus* | XP_024425698.1 |
| Fruit bat | *Rousettus leschenaultii* | BAF50705.1 |
| Indian false vampire bat | *Megaderma lyra* | QKE49998.1 |
| Greater horseshoe bat | *Rhinolophus ferrumequinum* | BAH02663.1 |
| Halcyon horseshoe bat | *Rhinolophus alcyone* | ALJ94035.1 |
| Intermediate horseshoe bat | *Rhinolophus affinis* | QMQ39240.1 |
| Japanese horseshoe bat | *Rhinolphus cornutus* | BCG67443.1 |
| Shamel's horseshoe bat | *Phinolophus shameli* | UBB59645.1 |
| Pearson's Horseshoe bat | *Rhinolophus pearsonii* | QKE49996.1 |
| Chinese rufous horseshoe bat | *Rhinolophus sinicus* | QMQ39219.1 |
| Big-eared Horseshoe bat | *Rhinolophus macrotis* | ADN93471.1 |
| Indian flying fox bat | *Pteropus giganteus* | XP_039729365.1 |
| Least Horseshoe bat | *Rhinolophus pusillus* | ADN93477.1 |

**Table S4. Detailed information of the isothermal titration calorimetry experiments.**

| Cell | [Cell] (µM) | Syringe | [Syr] (µM) | Injections | N (sites) | KD (nM) | ∆H (kcal/mol) | ∆G (kcal/mol) | -T∆S (kcal/mol) |
| --- | --- | --- | --- | --- | --- | --- | --- | --- | --- |
| CEACAM6 | **8.65** | **CcCoV\|2B** | **96.3** | **13** | **0.936** | **87.6** | **-11.3** | **-9.63** | **1.71** |
| CEACAM6 | **9.36** | **CcCoV\|2B** | **9.9.1** | **19** | **0.886** | **55.7** | **-10.8** | **-9.9** | **0.915** |
| CEACAM6 | **8.36** | **CcCoV\|2B** | **86.0** | **13** | **0.91** | **67.2** | **-11.7** | **-9.79** | **1.88** |
| CEACAM6 | **9.36** | **CcCoV\|KY43** | **107** | **13** | **0.967** | **202** | **-11** | **-9.13** | **1.83** |
| CEACAM6 | **8.36** | **CcCoV\|KY43** | **100** | **13** | **0.983** | **337** | **-12.6** | **-8.83** | **3.79** |
| CEACAM6 | **8.36** | **CcCoV\|KY43** | **101** | **13** | **0.886** | **274** | **-13** | **-8.96** | **4.05** |
| CEACAM5 | **6.53** | **CcCoV\|KY43** | **100** | **13** | **–** | **–** | **–** | **–** | **–** |
| CEACAM5 | **9.36** | **CcCoV\|KY43** | **96.83** | **13** | **–** | **–** | **–** | **–** | **–** |

**Table S5. Crystallographic data collection and refinement statistics.**

|  | CcCoV KY43 RBD + CEACAM6 | CcCoV-2B RBD + CEACAM6 |
| --- | --- | --- |
| **Data collection** |  |  |
| Space group | *P*2_1_ | *P*2_1_2_1_2_1_ |
| Cell dimensions |  |  |
| *a*, *b*, *c* (Å) | 70.01, 78.80, 82.34 | 47.55, 76.86, 204.98 |
| α, β, γ (°) | 90, 88.64, 90 | 90, 90, 90 |
| Resolution (Å) | 82.33–3.01^a^ (3.39–3.01)* | 61.38–2.99^b^ (3.31–2.99)* |
| *R*_merge_ | 0.196 (1.259) | 0.226 (1.392) |
| *I* / σ*I* | 5.4 (1.6) | 6.4 (1.6) |
| *CC_1/2_* | 0.994 (0.583) | 0.974 (0.844) |
| Completeness (spherical, %) | 48.5 (8.3) | 59.2 (12.0) |
| Completeness (ellipsoidal, %) | 88.9 (58.3) | 90.2 (73.4) |
| Redundancy | 6.2 (6.4) | 12.2 (12.7) |
|  |  |  |
| **Refinement** |  |  |
| Resolution (Å) | 82.32–3.01 | 61.49–2.99 |
| No. reflections | 8687 | 9371 |
| *R*_work_ / *R*_free_ | 0.256/0.317 | 0.258/0.300 |
| No. atoms |  |  |
| Protein | 3200 | 3104 |
| Carbohydrate | 140 | 168 |
| *B*-factors |  |  |
| Protein | 78.0 | 81.1 |
| Carbohydrate | 85.3 | 105.9 |
| R.m.s. deviations |  |  |
| Bond lengths (Å) | 0.005 | 0.003 |
| Bond angles (°) | 1.049 | 0.792 |

*Values in parentheses are for highest-resolution shell.

^a^Diffraction limit was 5.85 Å in direction 0.781 a* + 0.625 c*

^b^Diffraction limit was 4.74 Å in direction 0.958 a* + 0.287 c*

**Table S6. Details of the residue at the contact interfaces between CcCoV RBDs and human CEACAM6.**

**Hydrogen bonds**

| CEACAM6 | CcCoV\|KY43 | Distance (Å) | CcCoV\|2B | Distance (Å) |
| --- | --- | --- | --- | --- |
| G75 N(amide) | T552 O(carbonyl) | 2.97 | T548 O(carbonyl) | 2.89 |
| S127 O(carbonyl) | Y603 Oη | 2.46 | Y599 Oη | 2.52 |
| L129 O(carbonyl) |  |  | R513 Nη1 | 2.84 |
| L129 O(carbonyl) | R517 Nη2 | 2.66 | R513 Nη2 | 2.84 |
| Q123 Oϵ | S555 Oγ | 2.67 | S551 Oγ | 2.59 |
| N131 N(amide) |  |  | S515 O(carbonyl) | 3.06 |
| E133 Oϵ | S519 Oγ | 2.67 | S515 Oγ | 2.66 |

**Hydrophobic interactions**

| CEACAM6 | CcCoV\|KY43 | CcCoV\|2B |
| --- | --- | --- |
| I63 side chain |  | Y599 side chain |
| Y65 peptide plane | W600 side chain |  |
| Y82 peptide plane | W600 side chain | W596 side chain |
| V83 side chain |  | W596 side chain |
| I125 side chain | W600 side chain | W596 side chain |
| D128 peptide plane | I605 side chain |  |
| L129 side chain | V556 side chain |  |
| L129 side chain |  | I594 side chain |
| L129 side chain | G599 peptide plane | G595 peptide plane |
| L129 side chain | Y603 side chain |  |
| L129 side chain | I605 side chain | I601 side chain |
| V130 side chain | T520 side chain | T516 side chain |

**Table S7. Accession numbers of the human CEACAM library.**

| Protein | GenBank accession number |
| --- | --- |
| CEACAM1 | NP_001703.2 |
| CEACAM3 | NP_001806.2 |
| CEACAM4 | NP_001808.2 |
| CEACAM5 | NP_004354.3 |
| CEACAM8 | NP_001807.2 |
| CEACAM20 | XP_011524731.1 |

**Table S8. General characteristics of the test sample populations.**

|  | Location | | | | | | |
| --- | --- | --- | --- | --- | --- | --- | --- |
|  | **Voi (n=32)** | **Ngao (n=39)** | **Hola (n=201)** | **Garsen (n=54)** | **Bura (n=42)** | **Total (n=368)** |  |
|  |  |  |  |  |  |  |  |
| Sex |  |  |  |  |  |  |  |
| Male | 29 (91%) | 33 (85%) | 197 (98%) | 48(89%) | 42 (100%) | 349 (95%) |  |
| Female | 3 (9%) | 6 (15%) | 4 (2%) | 6 (11%) | 0 (0%) | 19 (5%) |  |
|  |  |  |  |  |  |  |  |
| Age in years |  |  |  |  |  |  |  |
| 15-24 | 12 (37%) | 21 (53%) | 50 (25%) | 26 (48%) | 30 (71%) | 139 (38%) |  |
| 25-34 | 4 (13%) | 11 (28%) | 76 (38%) | 22 (40%) | 12 (29%) | 125 (34%) |  |
| 35-44 | 7 (22%) | 5 (14%) | 49 (24%) | 1(2%) | 0 (0%) | 62 (17%) |  |
| 45-54 | 4 (13%) | 2 (5%) | 20 (10%) | 2 (4%) | 0 (0%) | 28 (7%) |  |
| 55-66 | 5 (15%) | 0 | 6 (3%) | 3 (6%) | 0 (0%) | 14 (4%) |  |

**Table S9. Accession numbers of the alphaCoV S reconstructing CcCoV local phylogeny.**

| ID | GenBank accession number |
| --- | --- |
| CcCoV\|2B | WWB00503 |
| CcCoV\|2A | WWB00496 |
| BtMsCoV/GS2013 | AIA62271 |
| btCoV/HB20 | WCC61872 |
| btRfCoV/ZJ20 | WCC61907 |
| btRfCoV/LN20 | WCC61886 |
| btRfCoV/Kudep | XLE35989 |
| btRsCoV/YN20 | WCC61836 |

**Table S10. Accession numbers of the CEACAM6 library**

| Common name | Scientific name | Genbank accession number |
| --- | --- | --- |
| Human | *Homo sapiens* | NP_002474.4 |
| Sumatran orangutan | *Pongo abelii* | XP_009230921.1 |
| Northern white-cheeked gibbon | *Nomascus leucogenys* | XP_030653003.1 |
| Olive baboon | *Papio anubis* | XP_009192843.1 |
| Macaque | *Macaca mulatta* | XP_014979566.2 |
| Tufted capuchin | *Sapajus apella* | XP_032105171.1 |
| Pale spear-nosed bat | *Phyllostomus discolor* | XP_028386169.1 |
| Greater horseshoe bat | *Rhinolophus ferrumequinum* | XP_032985312.1 |
| Great roundleaf bat | *Hipposideros armiger* | XP_019490169.1 |
| Egyptian fruit bat | *Rousettus aegyptiacus* | XP_036088268.1 |
| Indian flying fox | *Pteropus giganteus* | XP_039705915.1 |
| Brandt’s bat | *Myotis brandtii* | XP_014386925.1 |
| Yuma myotis | *Myotis yumanensis* | XP_070254596.1 |
| Daubenton’s bat | *Myotis daubentonii* | XP_059522338.1 |
| Big brown bat | *Eptesicus fuscus* | XP_054566002.1 |
| Natal long-fingered bat | *Miniopterus natalensis* | XP_016064669.1 |
| Cat | *Felis catus* | XP_044902081.1 |
