# Supplementary material for: Heart-nosed bat alphacoronaviruses use human CEACAM6 to enter cells
Source: Nature. 2026 Apr 22;653(8113):180–9. doi: 10.1038/s41586-026-10394-x (PMC13149331; doi:10.1038/s41586-026-10394-x)
Supplement: Supplementary file 2 — Reporting Summary [file 41586_2026_10394_MOESM2_ESM.pdf]

nature portfolio

Corresponding author(s): Dalan Bailey, Stephen Graham

Last updated by author(s): Mar 3, 2026

Reporting Summary

Nature Portfolio wishes to improve the reproducibility of the work that we publish. This form provides structure for consistency and transparency in reporting. For further information on Nature Portfolio policies, see our Editorial Policies and the Editorial Policy Checklist.

Statistics

For all statistical analyses, confirm that the following items are present in the figure legend, table legend, main text, or Methods section.

n/a	Confirmed	
☐	☒	The exact sample size (*n*) for each experimental group/condition, given as a discrete number and unit of measurement
☐	☒	A statement on whether measurements were taken from distinct samples or whether the same sample was measured repeatedly
☐	☒	The statistical test(s) used AND whether they are one- or two-sided<br>*Only common tests should be described solely by name; describe more complex techniques in the Methods section.*
☐	☒	A description of all covariates tested
☐	☒	A description of any assumptions or corrections, such as tests of normality and adjustment for multiple comparisons
☐	☒	A full description of the statistical parameters including central tendency (e.g. means) or other basic estimates (e.g. regression coefficient) AND variation (e.g. standard deviation) or associated estimates of uncertainty (e.g. confidence intervals)
☐	☒	For null hypothesis testing, the test statistic (e.g. *F*, *t*, *r*) with confidence intervals, effect sizes, degrees of freedom and *P* value noted<br>*Give P values as exact values whenever suitable.*
☐	☒	For Bayesian analysis, information on the choice of priors and Markov chain Monte Carlo settings
☒	☐	For hierarchical and complex designs, identification of the appropriate level for tests and full reporting of outcomes
☐	☒	Estimates of effect sizes (e.g. Cohen's *d*, Pearson's *r*), indicating how they were calculated

Our web collection on statistics for biologists contains articles on many of the points above.

Software and code

Policy information about availability of computer code

Data collection	Viral sequences used for this study were obtained from https://www.bv-brc.org/view/Virus/10239.<br><br>Diffraction data were recorded from single crystals on Diamond beamline I24 at 20 keV using an Eiger2 9M detector (CcCoV|KY43 complex) or on beamline I04 at 13 keV using an Eiger2 X 16M detector (CcCoV|2B complex).<br><br>Human population data was obtained from the Kenya National Bureau of Statistics (KNBS, 2019, https://www.knbs.or.ke/download/2019-kenya-population-and-housing-census-volume-iii-distribution-of-population-byage-sex-and-administrative-units/) based on the 2019 national census.
Data analysis	R 4.4.1 (ttps://www.r-project.org/) packages were downloaded from https://cran.r-project.org/web/packages/available_packages_by_name.html.<br><br>For protein structure visualization, PyMol version 2.4.0a0 (https://pymol.org/) or ChimeraX were used. Structural models were solved by molecular replacement using PHASER, and manually improved using ISOLDE and COOT software.<br>Structure interfaces were analysed using PDBePISA.<br><br>Sequence alignments were obtained using MAFFT 7.526. Molecular clock calibration was performed in BEAST 1.10.5. Highest Independent Posterior Subtree Reconstructed (HIPSTR)81 trees were constructed in TreeAnnotator.<br><br>For the design of primers used for site-directed mutagenesis, the Agilent online tool was used: https://www.agilent.com/store/primerDesignProgram.jsp

For the design of shRNA used for knock-down of human CEACAM6, the web portal of the Genetic Perturbation Consortium (Broad Institute, https://portals.broadinstitute.org/gpp/public/seq/search) was used.

Publicly available single cell RNA-sequencing (scRNA-seq) data from the Human Protein Atlas were downloaded from https://www.proteinatlas.org/humanproteome/single+cell/single+cell+type/data#datasets.
Lung Cell Atlas (HLCA v1.0) dataset for normal human lungs samples from (https://data.humancellatlas.org/hca-bio-networks/lung/atlases/lung-v1-0). Processed cell-type annotations and expression matrices were loaded into Seurat (v.4.3.3.).

For data and statistical analysis, GraphPad Prism9 was used. For analysis of relative changes in pseudotype entry, significance was quantified using the Python3 module statsmodels (https://www.statsmodels.org/).

For data visualization, Adobe Illustrator was used.

For manuscripts utilizing custom algorithms or software that are central to the research but not yet described in published literature, software must be made available to editors and reviewers. We strongly encourage code deposition in a community repository (e.g. GitHub). See the Nature Portfolio guidelines for submitting code & software for further information.

Data

Policy information about availability of data

All manuscripts must include a data availability statement. This statement should provide the following information, where applicable:

- Accession codes, unique identifiers, or web links for publicly available datasets
- A description of any restrictions on data availability
- For clinical datasets or third party data, please ensure that the statement adheres to our policy

Atomic coordinates, structure factors and protein sequences have been deposited to the Protein DataBank (PDB). Accession numbers are PDB: 9RCS and 9RCU for human CEACAM6 in complex with CcCoV|KY43 RBD and CcCoV|2B RBD, respectively. In addition to the structures determined in this study, the following previously experimentally determined 3D structures have been included in this article: CEACAM6 homodimer (PDB:4Y8A), CEACAM6-CEACAM8 heterodimer (PDB:4YIQ), btCoV/MOW15-22 (PDB: 9C6O), hCoV/SARS2, PDB: 6M0J; hCoV/SARS1, PDB: 2AJF4 NeoCoV, PDB: 7WPO; btCoV/HKU5, PDB: 9D32; btCoV/KY72, PDB: 8K4U; btCoV/PRD-0038, PDB: 8U0T, hCoV/NL63 (PDB: 3KBH), hCoV/229E (PDB: 6ATK), canine CoV (CCoV strain HuPn2018, PDB: 7U0L) and porcine coronavirus PRCV (PDB: 4F5C), PDCoV (PDB: 7VPQ), MHV S trimer in complex with murine CEACAM1 (PDB:6VSJ). All sequence data analysed were sourced from public databases, as described in Materials and Methods. These, along with the raw, when necessary, analysed data presented in this article, have been deposited in Zenodo and made freely accessible at the following link: 10.5281/zenodo.17951484.
Human population data was obtained from the Kenya National Bureau of Statistics (KNBS, 2019, https://www.knbs.or.ke/download/2019-kenya-population-and-housing-census-volume-iii-distribution-of-population-byage-sex-and-administrative-units/) based on the 2019 national census. Viral sequences used for this study were obtained from https://www.bv-brc.org/view/Virus/10239. Publicly available single cell RNA-sequencing (scRNA-seq) data from the Human Protein Atlas were downloaded from https://www.proteinatlas.org/humanproteome/single+cell/single+cell+type/data#datasets.
Lung Cell Atlas (HLCA v1.0) dataset for normal human lungs samples from (https://data.humancellatlas.org/hca-bio-networks/lung/atlases/lung-v1-0).
The raw human data shown in the manuscript are subject to controlled access because they are the subject of ongoing work and will be made available on request to the corresponding author and approval by the Data Governance Committee at the KEMRI-Wellcome Trust Research Programme. De-identified data has been published on the Harvard dataverse server https://doi.org/10.7910/DVN/XSGOOF.

Research involving human participants, their data, or biological material

Policy information about studies with human participants or human data. See also policy information about sex, gender (identity/presentation), and sexual orientation and race, ethnicity and racism.

Reporting on sex and gender	yes
Reporting on race, ethnicity, or other socially relevant groupings	yes
Population characteristics	The study population is made up of blood donors. The Kenya National Blood Transfusion Services guidelines define eligible donors as individuals aged 16-65 years, weighing >=50kg, with hemoglobin of 12.5g/dl, a normal blood pressure (systolic 120-129 mmHg and diastolic BP of 80-89 mHg), a pulse rate of 60-100 beats per minute and without any history of illness in the past 6 months.
Recruitment	The study population was recruited as anonymized residual samples from consecutive donor units submitted to the regional centres for transfusion compatibility-testing and infection screening. Since blood donors are restricted to those 16-65 years old, they are not representative of a population sample of all ages which may introduce a selection bias. In addition, the eligibility criteria for blood donation may select for more healthy members of the population which may lead to underestimation of KY43 antibody prevalence
Ethics oversight	The Scientific and Ethics Review Unit of the Kenya Medical Research Institute gave ethical approval

Note that full information on the approval of the study protocol must also be provided in the manuscript.

Field-specific reporting

Please select the one below that is the best fit for your research. If you are not sure, read the appropriate sections before making your selection.

☒ Life sciences ☐ Behavioural & social sciences ☐ Ecological, evolutionary & environmental sciences

For a reference copy of the document with all sections, see nature.com/documents/nr-reporting-summary-flat.pdf

Life sciences study design

All studies must disclose on these points even when the disclosure is negative.

Sample size	Among the total number of viral S protein available, a subset of 40 was used for this study. This number is based on the screening and funding capabilities associated to the study. The selected 40-sequence S panel (~1.5% of the full dataset) captured 53.4% of the total phylogenetic diversity, substantially exceeding size-matched random panels (13.7±3.2%; 10,000 permutations; ~3.9-fold enrichment; empirical $p<0.0001$). Once we identified the receptor of an alphacoronavirus S, we focused on validating its interaction. For analysis of human sera, 368 samples were analysed, based on the availability and the donor recruited.
Data exclusions	No data was excluded
Replication	Initial receptor screening was performed in technical triplicates, with two independent set of pseudoparticles (biological duplicates). Positive results were validated with at least 3 biological replicates, in technical triplicates.
Randomization	Human samples are convenient residual blood donor samples from the regional centres of transfusion, in the regions where CcCoV|KY43 was identified. Since blood donors are restricted to those 16-65 years old, they are not representative of a population sample of all ages which may introduce a selection bias. This is based on access to population.
Blinding	The population was recruited as anonymized residual samples. De-identified data has been published on the Harvard dataverse server https://doi.org/10.7910/DVN/XSGOOF.

Reporting for specific materials, systems and methods

We require information from authors about some types of materials, experimental systems and methods used in many studies. Here, indicate whether each material, system or method listed is relevant to your study. If you are not sure if a list item applies to your research, read the appropriate section before selecting a response.

Materials & experimental systems

n/a	Involved in the study	
☐	☒	Antibodies
☐	☒	Eukaryotic cell lines
☒	☐	Palaeontology and archaeology
☒	☐	Animals and other organisms
☒	☐	Clinical data
☒	☐	Dual use research of concern
☒	☐	Plants

Methods

n/a	Involved in the study	
☒	☐	ChIP-seq
☐	☒	Flow cytometry
☒	☐	MRI-based neuroimaging

Antibodies

Antibodies used	V5 Tag Monoclonal Antibody, PE conjugated (Invitrogen, clone TCM5)<br>FLAG Tag (DYKDDDDK) Antibody, APC conjugated (Miltenyi Biotec, clone REA216)<br>His tag antibody, APC conjugated (Miltenyi Biotec, clone GG11-8F3.5.1)<br>antibodies against human CEACAM6 (ThermoFisher, clones B6.2 and 439424)<br>HIV capsid p24 (Abcam, clone 5)<br>HA tag, unconjugated (Cell Signaling Technology, clone 6E2)<br>HA tag, PE conjugated (Miltenyi Biotec, clone GG8-1F3.3.1)<br>Flag tag, PE conjugated (Biolegend, clone L5)<br>Goat anti-mouse IgG(H+L), FITC conjugated (Invitrogen, polyclonal)<br>Goat anti-mouse IgG (H+L) DyLight 680 (Invitrogen, ployclonal)
Validation	V5 Tag Monoclonal Antibody, PE conjugated (Invitrogen, clone TCM5) - validation for flow cytometry:<br>https://www.thermofisher.com/antibody/product/V5-Tag-Antibody-clone-TCM5-Recombinant-Monoclonal/740058M<br>HEK-293E cells were transiently transfected with V5-H3-His construct (pink histogram) or left untransfected (blue histogram). Cells were fixed and permeabilized using the Foxp3 / Transcription Factor Staining Buffer Set (Product # 00-5523-00) and then stained intracellularly with 0.25 µg of V5 Tag Recombinant Mouse Monoclonal Antibody (TCM5), (Product # 740058M), followed by Goat anti-Mouse IgG (H+L), Superclonal™ Recombinant Secondary Antibody, Alexa Fluor™ Plus 647 (Product # A55060, 1:500). Viable cells were used for analysis, as determined by LIVE/DEAD™ Fixable Violet Dead Cell Stain Kit (Product # L34955). The flow cytometry data

was acquired using Attune™ NxT Flow Cytometer (Product # A29004).

FLAG Tag (DYKDDDDK) Antibody, APC conjugated (Miltenyi Biotec, clone REA216) - validation for flow cytometry:
https://www.miltenyibiotec.com/GB-en/products/dykddddk-antibody-reafinity-rea216.html#conjugate=vio-b515:size=100-tests-in-200-ul
293HEK cells stably transfected with FLAG®-tagged human Argonaute 1 protein were stained intracellularly with Anti-DYKDDDDK antibodies and analyzed by flow cytometry using the MACSQuant® Analyzer.

His tag antibody, APC conjugated (clone GG11-8F3.5.1, Miltenyi Biotec) - validation for flow cytometry:
https://www.miltenyibiotec.com/GB-en/products/his-antibody-gg11-8f3-5-1.html#conjugate=fitc:size=100-tests-in-200-ul
Surface staining follow protocol (https://www.miltenyibiotec.com/GB-en/applications/all-protocols/cell-surface-flow-cytometry-staining-protocol-pbs-edta-bsa-1-50.html)

Antibodies against human CEACAM6 (ThermoFisher, clones B6.2 and 439424) - validation for western blot:
Lysates of HEK293T transfected with a plasmid encoding for human CEACAM6 after 24 hours. Expression was analysed by sodium dodecyl sulfate polyacrylamide gel electrophoresis (SDS-PAGE). Separated proteins were transferred onto a 0.45 µm nitrocellulose membrane (Cytiva), blocked in PBS supplemented with 0.05% TWEEN-20 and 5% (w/v) unskimmed milk powder, and incubated with mouse monoclonals overnight at 4C. The following day, goat anti-mouse DyLight 680 (Invitrogen, 1:10000) was used to probe primary antibodies, and signals detected with an Odyssey DLx imaging system (LI-COR Biosciences). Control lysate non-transfected, or transfected with non-relevant proteins, were run in parallel to assess specificity.
references citing the use of the antibody: DOI: 10.14348/molcells.2020.2230

HIV capsid p24 (clone 5, Abcam) - validation for Western blot:
references citing the use of the antibody: DOI: 10.1016/j.isci.2022.105016, DOI: 10.1021/acs.biochem.3c00109, DOI: 10.1186/s12964-024-01795-4

HA tag, unconjugated (Cell Signaling Technology, clone 6E2) - validation for Western blot:
https://www.cellsignal.com/products/primary-antibodies/ha-tag-6e2-mouse-monoclonal-antibody/2367
references citing the use of the antibody: DOI: 10.1038/s41586-025-09727-z, DOI: 10.1038/s41467-025-66120-0, DOI: 10.1038/s41586-025-09768-4

HA tag, PE conjugated (Miltenyi Biotec, clone GG8-1F3.3.1) - validation for flow cytometry:
https://www.miltenyibiotec.com/GB-en/products/ha-antibody-gg8-1f3-3-1.html#conjugate=apc:size=100-tests-in-200-ul
Surface staining follow protocol (https://www.miltenyibiotec.com/GB-en/applications/all-protocols/cell-surface-flow-cytometry-staining-protocol-pbs-edta-bsa-1-50.html)

Flag tag, PE conjugated (Biolegend, clone L5) - validation for flow cytometry:
https://www.biolegend.com/en-gb/products/purified-anti-dykddddk-tag-antibody-4905
references citing the use of the antibody: DOI: 10.1074/jbc.M109.071696, DOI: 10.1016/j.molcel.2018.09.029, DOI: 10.1016/j.celrep.2022.111582, DOI: 10.1016/j.cell.2022.11.033

Goat anti-mouse IgG(H+L), FITC conjugated (Invitrogen, polyclonal) - validation for Western blot:
https://www.thermofisher.com/antibody/product/Goat-anti-Mouse-IgG-H-L-Cross-Adsorbed-Secondary-Antibody-Polyclonal/31541
references citing the use of the antibody: DOI: 10.1096/fj.201900260R, DOI: 10.1007/978-1-60761-063-2_1

Goat anti-mouse IgG (H+L) DyLight 680 (Invitrogen, ploycloncal) - validation for Western blot:
https://www.thermofisher.com/antibody/product/Goat-anti-Mouse-IgG-H-L-Secondary-Antibody-Polyclonal/35518
references citing the use of the antibody: DOI: 10.1038/s44318-025-00616-9, DOI: 10.1371/journal.pbio.3003320, DOI: 10.1371/journal.ppat.1013410

Eukaryotic cell lines

Policy information about cell lines and Sex and Gender in Research

Cell line source(s)	HEK239T - ATCC (CRL-3216), THP-1 - ATCC (TIB-202), Calu-3 - ATCC (HTB-55), Caco-2 - ATCC (HTB-37), HeLa - ATCC (CCL-2), Expi293 - ThermoFisher, HuH7 were kindly provided by dr. Jane McKeating (University of Oxford), LCL cells (patient B cells immortalised with EBV) were kindly provided by dr. Claire Shannon Lowe (University of Birmingham).
Authentication	Cell lines were not authenticated
Mycoplasma contamination	All cell lines tested negative for mycoplasma contamination
Commonly misidentified lines (See ICLAC register)	*Name any commonly misidentified cell lines used in the study and provide a rationale for their use.*

Plants

Seed stocks	n/a
Novel plant genotypes	n/a
Authentication	n/a

Flow Cytometry

Plots

Confirm that:

☒ The axis labels state the marker and fluorochrome used (e.g. CD4-FITC).

☒ The axis scales are clearly visible. Include numbers along axes only for bottom left plot of group (a 'group' is an analysis of identical markers).

☒ All plots are contour plots with outliers or pseudocolor plots.

☒ A numerical value for number of cells or percentage (with statistics) is provided.

Methodology

Sample preparation	cells were resuspended in PBS, fixed using 2% paraformaldehyde for 20 min at 4°C and permeabilized using PBS supplemented with 0.5% Triton X100 for 5 min at 4°C. After washing, cells were incubated for 1 h with an anti-tag antibody. Cells were finally washed three times before analysis.
Instrument	MACSQuant Analyzer 10 cytometer
Software	FlowJo (BD Biosciences)
Cell population abundance	For PE staining:<br>Total cell count: around 18K cells/sample.<br><br>For APC staining:<br>Total cell count: around 55K cells/sample<br><br>For FITC staining:<br>Total cell count: around 25K cells/sample.
Gating strategy	Gating was performed on the negative controls (cell transfected with a vector not expressing the receptor and stained with the anti-tag antibody). Positive staining was defined above 10^3 of PE-A/APC-A/FITC-A signals.

☒ Tick this box to confirm that a figure exemplifying the gating strategy is provided in the Supplementary Information.
